# ATP1A1/BCL2L1 predicts the response of myelomonocytic and monocytic acute myeloid leukemia to cardiac glycosides

**DOI:** 10.1038/s41375-023-02076-8

**Published:** 2023-10-30

**Authors:** Claudia Cerella, Sruthi Reddy Gajulapalli, Anne Lorant, Deborah Gerard, Florian Muller, Yejin Lee, Kyung Rok Kim, Byung Woo Han, Christo Christov, Christian Récher, Jean-Emmanuel Sarry, Mario Dicato, Marc Diederich

**Affiliations:** 1 Laboratoire de Biologie Moléculaire et Cellulaire du Cancer (LBMCC), Fondation Recherche sur le Cancer et les Maladies du Sang, Pavillon 2, 6A rue Barblé, L-1210 Luxembourg, Luxembourg; 2 Research Institute of Pharmaceutical Sciences & Natural Products Research Institute, College of Pharmacy, Seoul National University, Seoul, 08826 Republic of Korea; 3 University of Lorraine, Service Commun de Microscopie, Nancy, France; 4 Cancer Research Center of Toulouse, UMR 1037 INSERM/ Université Toulouse III-Paul Sabatier, 2 avenue Hubert Curien, Oncopôle, 31037 Toulouse, France

**Keywords:** Acute myeloid leukaemia, Acute myeloid leukaemia, Apoptosis

## Abstract

Myelomonocytic and monocytic acute myeloid leukemia (AML) subtypes are intrinsically resistant to venetoclax-based regimens. Identifying targetable vulnerabilities would limit resistance and relapse. We previously documented the synergism of venetoclax and cardiac glycoside (CG) combination in AML. Despite preclinical evidence, the repurposing of cardiac glycosides (CGs) in cancer therapy remained unsuccessful due to a lack of predictive biomarkers. We report that the ex vivo response of AML patient blasts and the in vitro sensitivity of established cell lines to the hemi-synthetic CG UNBS1450 correlates with the ATPase Na^+^/K^+^ transporting subunit alpha 1 (ATP1A1)/BCL2 like 1 (BCL2L1) expression ratio. Publicly available AML datasets identify myelomonocytic/monocytic differentiation as the most robust prognostic feature, along with core-binding factor subunit beta (*CBFB*), lysine methyltransferase 2A (*KMT2A*) rearrangements, and missense Fms-related receptor tyrosine kinase 3 (*FLT3*) mutations. Mechanistically, BCL2L1 protects from cell death commitment induced by the CG-mediated stepwise triggering of ionic perturbation, protein synthesis inhibition, and MCL1 downregulation. In vivo, CGs showed an overall tolerable profile while impacting tumor growth with an effect ranging from tumor growth inhibition to regression. These findings suggest a predictive marker for CG repurposing in specific AML subtypes.

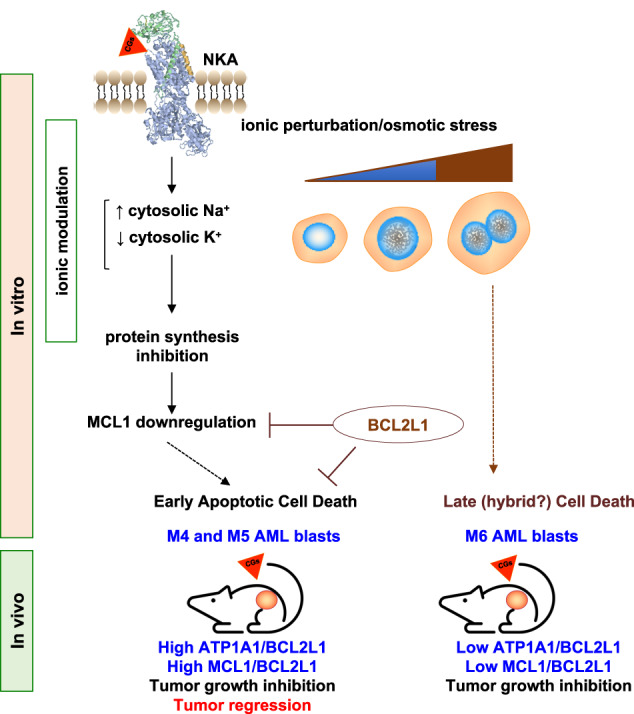

## Introduction

Myelomonocytic and monocytic acute myeloid leukemia (AML) has been frequently associated with venetoclax (VEN) resistance. In a multivariate analysis, the monocytic maturation state was the only predictor of a refractory response to VEN + azacytidine (VEN + AZA) [1]. An integrated analysis aiming at identifying novel predictive biomarkers of VEN efficacy and suitable combinatorial regimens identified the ex vivo resistance of myelomonocytic/monocytic AML (French-American-British (FAB) classification M4/M5 or CD14^+^ phenotype) blasts against VEN [2]. An ex vivo phenotype-based drug screening revealed an association between the increasing maturation state of AML blasts and VEN resistance [3]. Furthermore, a monocyte-associated gene signature based on a Bayesian multisource regression predicted resistance of AML blasts to BCL2 inhibitors VEN and navitoclax [4]. Identifying and targeting myelomonocytic/monocytic clones from the diagnosis emerged as an important therapeutic strategy [5] conditioned by routine clinical immunophenotyping of monocytic clones by CD14, CD68, FCGR1A (CD64), ITGAM (CD11b) markers [1], stand-alone or incorporated in more elaborated monocytic signatures [2, 4, 6, 7]. Although multiple ex vivo studies documented an upfront resistance of AML blasts with monocytic differentiation to VEN + AZA, recent clinically oriented investigations question the robustness of monocytic differentiation as a predictor of VEN-based therapy response in clinical settings. The percentage of immature monocytes or the frequency of CD64/CD11b^+^ monocytic blasts did not predict therapy response. Moreover, these characteristics were not enriched in refractory/relapsed AML patients. Indeed leukemic stem cell (LSC) resistance is considered to determine therapy outcome [8, 9]. Recently, VEN-based therapies revealed the existence of a novel monocytic LSC (m-LSC) promoting a monocytic AML progression [10]. Defining markers more closely associated with emerging diversified monocytic-like phenotypes could be critical for predicting upfront therapy resistance.

The anti-apoptotic protein apoptosis regulator B-cell lymphoma-2 (BCL2) family member (MCL1) contributes to the resistance to VEN plus hypomethylating agents (HMAs) [11]. MCL1 inhibition or downregulation re-sensitizes AML blasts to VEN *via* mechanisms including mitochondrial priming and the impairment of the reliance of LSCs on lipid metabolism [12]. Therefore, targeting MCL1 function/expression is a strategy pursued by investigational studies and clinical trials. Notably, monocytic AML blasts express higher MCL1 and lower BCL2 levels than primitive AML clones [1]. This BCL2 protein expression pattern correlates with MCL1/BCL2 co-dependency [13]. MCL1 but not BCL2 protein dependency also characterizes the newly postulated m-LSCs [10]. While there is robust evidence of opposite BCL2 and MCL1 expression levels in primitive vs. more mature blasts, differential BCL2-like 1 (BCL2L1) expression in monocytic AML blasts remains less evident [9]. These observations contrast with the dependency of erythroid/megakaryocytic blasts on BCL2L1 overexpression [14].

We identified cardiac glycosides (CGs) as post-transcriptional MCL1 inhibitors, as MCL1 was downregulated before caspase cleavage in multiple cancer cell types [15, 16]. BCL2 was not or barely impacted, even at doses beyond the IC50. The modulation of BCL2L1 was cell-type dependent and occurred either simultaneously or after caspase cleavage. BCL2 overexpression in BCL2/MCL1 co-expressing AML cells did not prevent CG-induced apoptotic cell death [16]. AML cell models expressing high levels of BCL2L1 maintained stable levels of this protein and displayed higher resistance to CG treatment [17]. These results were also observed in solid tumor cell types with similar patterns of BCL2 protein expression [16]. Early downregulation of MCL1 enhances CG-induced cytocidal effects and synergizes with VEN in AML cell models and patient blasts [16, 17].

The sodium/potassium ATPase (NKA) is the established cellular target of CGs, used against congestive heart diseases [18]. A physical interaction between the monocytic marker CD14 and the ATP1A1 isoform of the NKA was recently described to modulate inflammatory pathways elicited by monocyte-derived cells, including macrophages [19]. This emerging evidence suggests that monocytic-like cells might rely more on ATP1A1 (and NKA) than other myeloid cell types to carry out cell type-specific biological functions and likely express higher levels of this NKA subunit.

We report that myelomonocytic and monocytic AML patient blasts express higher levels of the ATPase Na^+^/K^+^ transporting subunit alpha 1 (ATP1A1) and lower levels of the anti-apoptotic BCL2L1. This expression pattern is consistent in adult and pediatric publicly available cohorts. The combined ATP1A1/BCL2L1 ratio is even more specifically associated with myelomonocytic and monocytic AML phenotypes. We show and mechanistically validate that the ATP1A1/BCL2L1 ratio improves CG sensitivity prediction. Considering the overall tolerable profile and the tumor growth impact observed in vivo, we suggest de novo or relapsed AML patients with myelomonocytic/monocytic phenotypes and genetic aberrations associated with this maturation state (i.e., *CBFB* and *KMT2A* rearrangements, and Fms-related receptor tyrosine kinase 3 (*FLT3*) missense mutations) [1] as potential candidates for combinatorial ATP1A1 targeting strategies by CGs.

## Methods

Ethical considerations, experimental protocols, cell lines, reagents, antibodies, and data analysis methods are available in Supplementary Data (Supplementary Methods).

### Healthy blood and patient samples

AML specimens were from a cohort described previously [17]. Healthy human umbilical cord blood (CB) CD34^+^ cells were collected as previously detailed [20]. Healthy adult donor buffy coats were used to isolate peripheral blood mononuclear cells (PBMCs) via density gradient centrifugation [21]. See Supplementary Methods for additional information about the source, authentication, and culture conditions of all cell models used in this study.

### Functional studies

Two *BCL2L1* siRNAs (Hs_BCL2L1_2 and Hs_BCL2L1_8, FlexiTube siRNA) and the control Allstar Negative siRNA were purchased from Qiagen (Venlo, The Netherlands). Mouse *Atp1a1* (Clone Id: 5321268) and human *ATP1A1* (Clone Id: 6065209) mammalian expression constructs were purchased from Horizon Discovery (Cambridge, UK).

### In vivo studies

All animal studies were performed according to the guidelines of the Institute of Animal Care and Use Committee of Seoul National University, Seoul, South Korea. The protocol was approved by the Committee of ethics of IACUC (SNU-190508-7-5). Their detailed description can be found in Supplementary Methods (Supplementary Data).

### Computational docking and bioinformatics analysis

The datasets and databases used in this manuscript are listed in Supplementary Table S17. Detailed information about computational studies (docking and bioinformatics) is in Supplementary Methods.

### Statistical analysis

Statistical analyses were performed with GraphPad Prism software (version 9.4.1; GraphPad, La Jolla, CA, USA), except for specific bioinformatics studies. See Supplementary Methods for additional information.

## Results

### Myelomonocytic and monocytic AML express higher levels of the ATP1A1/BCL2L1 ratio

**Fig. 1:**
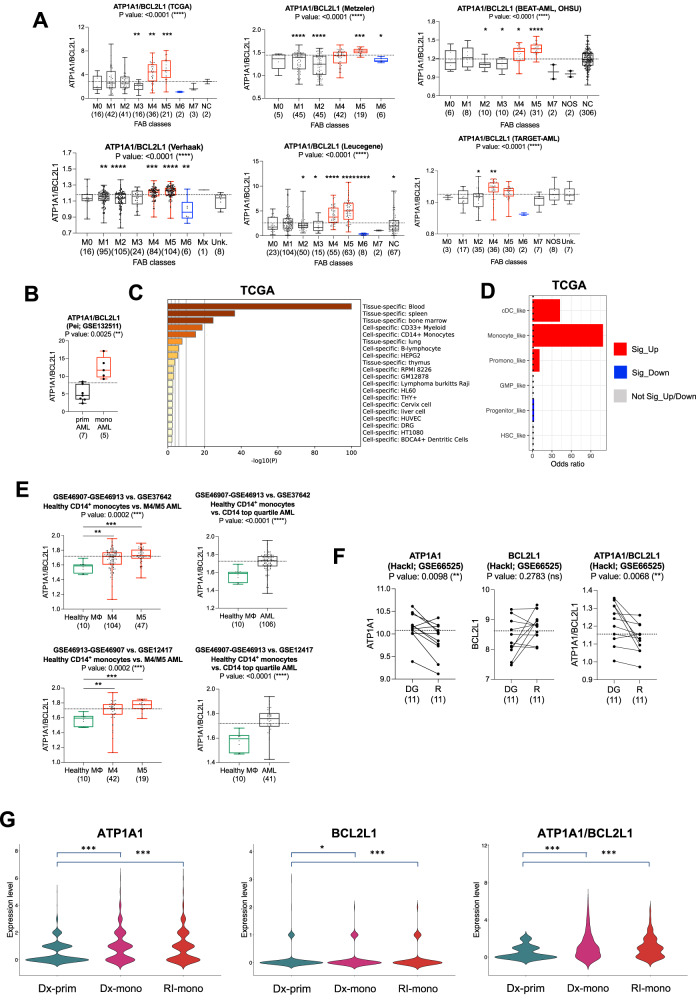
Myelomonocytic and monocytic AML express higher ATP1A1/BCL2L1 levels. ATP1A1/BCL2L1 in AML with different maturation states, determined by **A** FAB classification; **B** immunophenotyping. **C** Enriched tissue/cell-specific gene patterns in the upregulated gene list of *ATP1A1*/*BCL2L1*^high^ TCGA (≥median). **D** Odds plot of the cell type enrichment in *ATP1A1*/*BCL2L1*^high^ TCGA patients. Genes in: red: significantly upregulated; blue: significantly downregulated; gray: not significant (up or downregulated). **E** *ATP1A1*/*BCL2L1* levels in CD14^+^ monocytes from healthy individuals (*N* = 10) vs. M4/M5 (*N* = 151 and *N* = 61) or *CD14* top quartile AML patient blasts (*N* = 106 and *N* = 41). **F** 11 diagnostic/relapse paired AML specimens after conventional chemotherapy [50]. **G** phenotypically primitive vs. monocytic de novo AML at the diagnosis vs. the relapsed clones in AML patient 12 (Pei et al. [1]). Kruskal–Wallis test to compare medians; Mann–Whitney test for comparisons between the median of each subgroup and the overall median (dashed line), or between two groups; *P* values: *<0.05, **<0.01, ***<0.001, ****<0.0001.

Myelomonocytic (FAB M4) and/or monocytic (FAB M5) AML subtypes show a higher *ATP1A1*/*BCL2L1* ratio (*ATP1A1*/*BCL2L1*^high^). Similarly, monocytic AML subtypes classified by flow cytometric immunophenotyping [1] show higher *ATP1A1*/*BCL2L1* levels than the primitive AML (Fig. 1A, B and Supplementary Fig. S1A–C). This trend was specific to the *ATP1A1* isoform of the catalytic alpha subunit of the NKA (Supplementary Fig. S1D–F). Monocytic markers were preferentially expressed in *ATP1A1*/*BCL2L1*^high^ AML patients (≥ median values; Supplementary Table S1). In line with these results, gene signatures associated with cell- or tissue-specific monocytes/macrophages were commonly enriched in the same patient subgroups (Fig. 1C, Supplementary Fig. S2A, B, and Supplementary Tables S2–4). We next determined whether ATP1A1/BCL2L1^high^ was enriched for one of the six AML cell type signatures previously defined by van Galen and colleagues [22], taking the top 50 upregulated genes of each cell type as a reference. The signatures of promonocyte-like, monocyte-like, and conventional dendritic (cDC)-like cells were significantly over-represented in *ATP1A1*/*BCL2L1*^high^ blasts (Fig. 1D, and Supplementary Fig. S2C).

Next, we investigated whether higher ATP1A1/BCL2L1 levels were specific to AML monocytic blasts. Healthy bone marrow (BM) mononuclear cells (Vizome; vizome.org/aml2 [23]) showed reduced *ATP1A1*/*BCL2L1* levels and even lower expression than M4/M5 AML blasts (Supplementary Fig. S3A). Additionally, we integrated the expression data from two independent AML cohorts (Herold, GSE37642 [24] and Metzeler GSE12417 [25]) with CD14^+^ monocytes isolated from healthy donors (Baldwin, GSE46907 and GSE46913 [26]). For this analysis, we selected M4/M5 AML blasts; alternatively, we stratified AML blasts by *CD14* expression. In all instances, AML blasts presented higher *ATP1A1*/*BCL2L1* levels (Fig. 1E and Supplementary Fig. S3B).

Next, to assess the association between ATP1A1/BCL2L1^high^ and clinical features other than the maturation state, we stratified AML patients of different AML cohorts according to their ATP1A1/BCL2L1 median expression. Chi-square and Fisher’s exact test (Supplementary Tables S5–9) confirmed *ATP1A1*/*BCL2L1*^*high*^ as a clinical feature associated with blast maturation. In addition, AML patients carrying missense driver mutations of *FLT3* (i.e., TKD) and genetic rearrangements of *CBFB* and *KMT2A* (ex-*MLL*) also exhibited the *ATP1A1*/*BCL2L1*^high^ phenotype (Supplementary Fig. S4). This preferential association was also observed when a limited number of patients carrying the mutation/rearrangement were available to reach the significance. For example, all five patients with FLT3 missense driver mutations in the pediatric TARGET-AML cohort belong to the ATP1A1/BCL2L1^high^ group (Supplementary Table S9); the rare adult TCGA AML patients with *KMT2A* rearrangements preferentially were also ATP1A1/BCL2L1^high^ (Supplementary Table S5).

AML patients relapsing from standard treatments show an enrichment of primitive leukemic clones [27, 28]. Vice versa, the outgrowth of pre-existing monocytic subclones contributed to resistance in VEN-treated AML patients [1]. We compared the ATP1A1/BCL2L1 expression level using RNA-seq data from 11 pairs of patient samples at diagnosis and relapse after conventional chemotherapy. We also investigated samples from one AML patient showing co-existing primitive and monocytic AML subclones at diagnosis and VEN-relapse. AML patients relapsing from conventional chemotherapy showed lower *ATP1A1*/*BCL2L1* levels (Fig. 1F). In contrast, the monocytic subclone from the VEN-relapsed patient showed a significantly higher *ATP1A1*/*BCL2L1* expression level than the co-existing primitive clones at diagnosis (Fig. 1G).

Overall, *ATP1A1*/*BCL2L1* expression is a common feature of myelomonocytic and monocytic AML clones and is preferentially associated with alterations frequently found in M4/M5 patients.

### AML blasts with ATP1A1/BCL2L1^high^ are more sensitive to the cytocidal potential of CGs

**Fig. 2:**
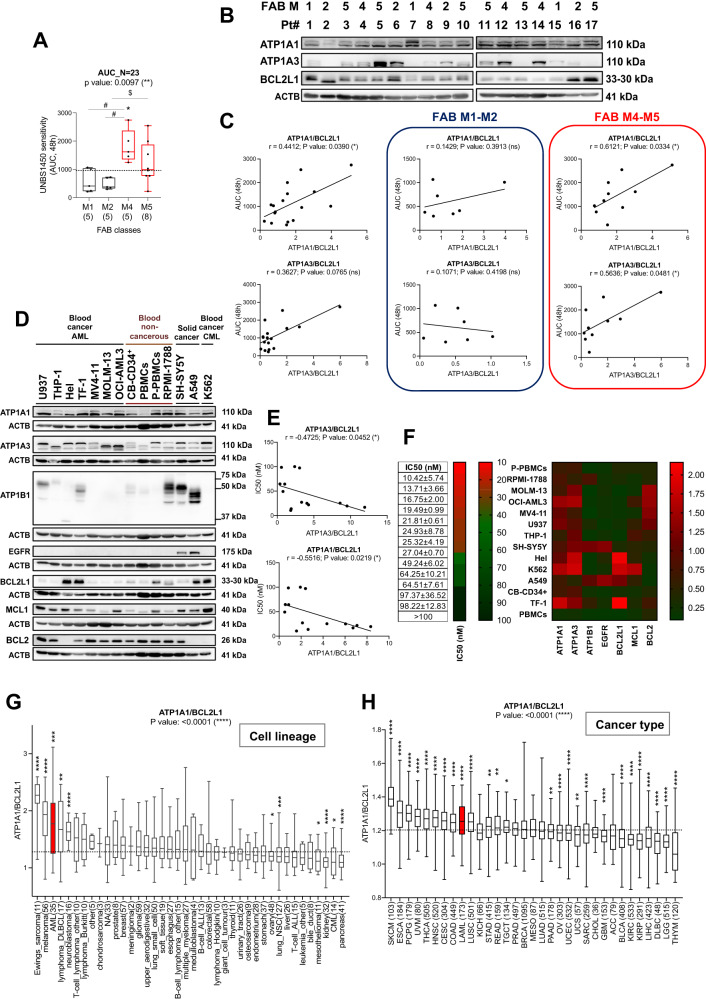
UNBS1450 sensitivity correlates with ATP1A1/BCL2L1 expression. **A** Ex vivo response of 23 de novo AML patient blasts to UNBS1450 according to their FAB subtype (AUC, area under the curve), and **B** western blot analysis of the indicated proteins [17]. The same membrane was probed for ATP1A1, ATP1A3, and BCL2L1. **C** Correlation analysis between UNBS1450 AUC values and ATP1A1/BCL2L1 and ATP1A3/BCL2L1 ratios estimated for the overall AML blast cohort (*N* = 17; non-parametric one-sided Spearman) or FAB M1-M2 (*N* = 6) vs. FAB M4-M5 subgroups (*N* = 11). **D** Western blot analysis of a panel of proteins on 14 cell models [45, 51]. Data are representative of three independent blots. **E** Correlation analysis between UNBS1450 IC50 mean values and ATP1A1/BCL2L1 and ATP1A3/BCL2L1 ratios in the selected cell models (non-parametric one-sided Spearman). **F** UNBS1450 IC50 values of the 14 cell models (mean of at least three independent experiments ±SD); heatmap visualization of the protein expression level (right). *ATP1A1*/*BCL2L1* levels across (**G**) 40 cancer cell lineages (CCLE; log2(RPKM) and (**H**) 33 primary cancer types (TCGA pan-cancer; log2(FPKM)–uq+1). Kruskal–Wallis test; comparisons between the median of each subgroup and the overall median (dashed line): Mann–Whitney test (*P* values: *<0.05, **<0.01, ***<0.001, ****<0.0001.

The CG UNBS1450 impacted AML cell lines and patient blasts as a single or combined treatment [16, 17]. In our original cohort of 23 de novo AML patients [17], the blast sensitivity to UNBS1450 was associated with the maturation state. The highest cytotoxicity was found in M4/M5 AML (Fig. 2A). Furthermore, ATP1A1/BCL2L1 expression significantly correlates with blast sensitivity to UNBS1450 in 17 AML patient samples retrieved from the original cohort (Rs: 0.4412, p-value: 0.0390). No significant correlations were found when the proteins were examined individually, or different ratios of ATP1A1/ATP1A3 with BCL2 proteins were calculated (Fig. 2B, C and Supplementary Fig. S5A–C). The correlation between ATP1A1/BCL2L1 expression and CG sensitivity became stronger when the analysis was restricted to FAB M4-M5 AML patient blasts (Rs: 0.6121, *p*-value: 0.0334; Fig. 2A). In this instance, we also found a significant correlation for the ATP1A3/BCL2L1 ratio (Rs: 0.5636, *p*-value: 0.0481). The FAB M1-M2 subgroup did not show significance (ATP1A1/BCL2L1, Rs: 0.1429, *p*-value: 0.3913; ATP1A3/BCL2L1, Rs: −0.1071, *p*-value: 0.4198). No significant correlations were found for both groups when the proteins were examined individually, or other ATP1A1/ATP1A3 and BCL2 protein ratios were calculated (Supplementary Fig. S5D).

A panel of 14 cell types, including AML and non-cancerous blood cell lineages, confirmed a significant inverse correlation between the impact of UNBS1450 on cell viability (IC50 values) and both ATP1A1/BCL2L1 and ATP1A3/BCL2L1 ratios with a stronger association in the instance of ATP1A1/BCL2L1 (Rs: −0.5516, *p*-value 0.0219, vs. Rs: −0.4725; *p*-value: 0.0452; Fig. 2D, E; Supplementary Fig. S5E, F).

The least sensitive cell model to UNBS1450 frequently showed elevated expression levels of the BCL2L1 protein (Fig. 2F). A lower ATP1A1/BCL2L1 ratio was a common feature of M6 AML patient blasts (Fig. 1A) and established cell lines (Supplementary Fig. S5G and Supplementary Table S10). AML was within the top three and the ninth highest *ATP1A1*/*BCL2L1* expressing types of cancer, respectively, across 40 cancer cell lineages (Cancer Cell Line Encyclopedia, CCLE, portal [29] and 33 tumor types (TCGA pan-cancer cohort [30]; Fig. 2G, H and Supplementary Tables S11, 12). *ATP1A1*/*BCL2L1* expression level was significantly above the average. Furthermore, AML consistently showed the lowest BCL2L1 expression level (Fig. 2G, H and Supplementary Fig. S6).

These results suggest that a multifactorial marker involving the NKA subunit ATP1A1 and the anti-apoptotic BCL2L1 is a more suitable predictor of the cell response to UNBS1450. M4/M5 AML are the most sensitive AML subtypes to CG treatment. In addition, CGs did not significantly affect the viability of monocytes from healthy donors (Supplementary Fig. S7).

### The cytotoxic potential of UNBS1450 is mediated by an ionic perturbation and is the consequence of an on-target NKA modulation

**Fig. 3:**
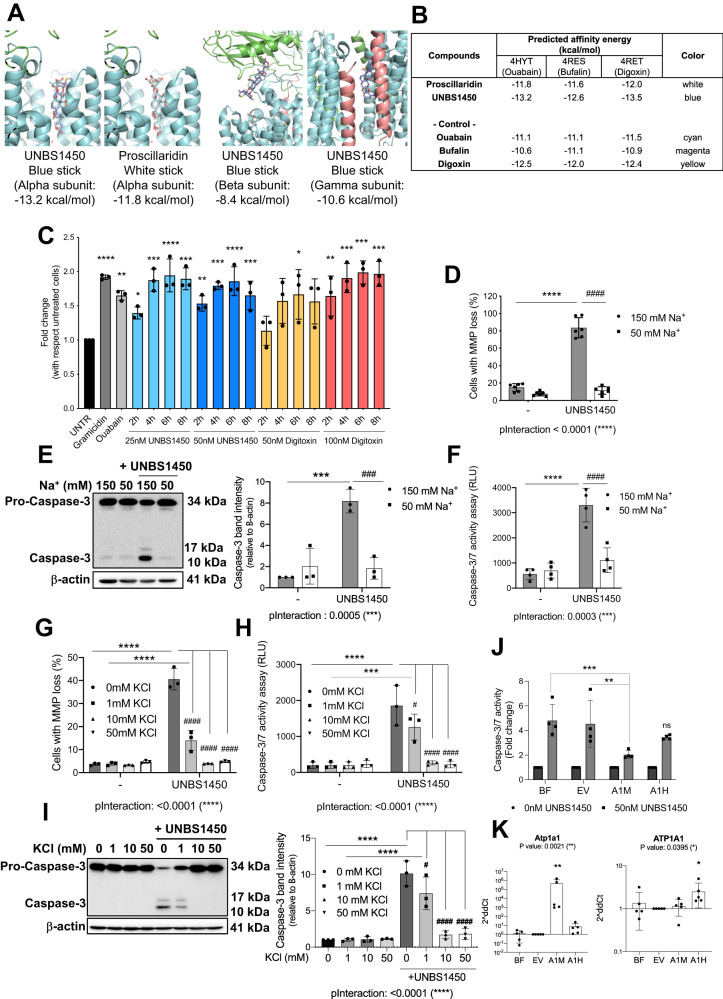
The cytotoxic potential of UNBS1450 is mediated by an ionic perturbation and is the consequence of an ON-target effect. **A** Docking orientation of UNBS1450 and proscillaridin on the crystal structure of the ATP1A1 (alpha) subunit and UNBS1450 in the crystal structure of ATP1B1 and ATP1C1 (beta and gamma) subunits of the NKA (PDB ID: 4HYT, 4RES, 4RET). **B** The predicted affinity energy (kcal/mol) of each CG. **C** Kinetic analysis of the intracellular Na^+^ levels in U937 cells treated with UNBS1450 (25 and 50 nM) or digitoxin (50 and 100 nM). Positive controls: Na^+^ ionophore gramicidin (10 µM) and ouabain (500 nM). Mean of *N* = 3 ± SD. One-way ANOVA, post-hoc: Dunnett; *P* values: *<0.05, **<0.01, ***<0.001, ****<0.0001; comparisons of each condition to the untreated sample. The cytotoxic potential of UNBS1450 (18 h) in U937 cells cultivated one h before treatment in salt-balanced modified media containing 150 or 50 mM Na^+^ on: **D** MMP loss (*N* = 6); **E** the caspase-3 (CASP-3) cleavage and quantification of the cleaved band intensity (right; *N* = 3); **F** the caspase-3/7 activity assay (*N* = 4). Significant statistical differences between untreated vs. UNBS1450-treated cells in the same type of medium (*) and UNBS1450-treated U937 cells in 150 mM vs. 50 mM medium (#): two-way ANOVA; post-hoc: Sidak; *P* values: */# < 0.05, **/## < 0.01, ***/### < 0.001, ****/#### < 0.0001). Same analyses on cell death modulation in U937 cells supplemented with 1, 10, or 50 mM KCl 30 min before adding UNBS1450: **G** MMP loss (*N* = 3); **H** caspase-3/7 activity (*N* = 3), and **I** CASP-3 cleavage with relative quantification of the cleaved band intensity (right; *N* = 3). Two-way ANOVA; post-hoc: Dunnett; *P* values: */# < 0.05, **/## < 0.01, ***/### < 0.001, ****/#### < 0.0001). **K** The Hel cell line was transfected with a plasmid bearing the cDNA of the murine *Atp1a1* or the human *ATP1A1*. **J** The caspase-3/7 activity (24 h treatment) with 50 mM UNBS1450 (*N* = 4; Two-way ANOVA, post-hoc: Sidak); **K** *Atp1a1* and *ATP1A1* mRNA levels monitored by RT-PCR at 48 h post-transfection, corresponding to the *t* = 0 h of UNBS1450 treatment (*N* = 5; Kruskal–Wallis; post-hoc: Dunn).

We then mechanistically validated the ATP1A1/BCL2L1 ratio as a marker of CG cell sensitivity. We focused first on the role of the NKA. Docking studies using the three different configurations of NKA crystalized with ouabain, bufalin, or digoxin [31, 32] documented that UNBS1450 was superior to any other CG for binding the alpha subunits in any configuration (Fig. 3A, B). The comparison between bufadienolide, proscillaridin, and UNBS1450 suggested a hydroxyl group relevant for increased binding affinity. UNBS1450 also showed an affinity for the gamma subunit (as good as the affinity of bufalin for the ouabain pose of the ATPase) and the beta subunit of the NKA (Fig. 3B).

The ionic function of the NKA is fundamental for cell homeostasis. NKA inhibition leads to an intracellular ionic perturbation with Na^+^ elicitation and K^+^ depletion [18, 33]. UNBS1450 time- and dose-dependently elicited intracellular Na^+^; digitoxin shared the same trend: Na^+^ elevation reached a plateau after four hours; the maximal intracellular Na^+^ increase was already evident at two hours at higher concentrations and comparable to ouabain and the Na^+^ ionophore gramicidin (Fig. 3C). To determine the modulatory role of the ionic perturbation on the cytotoxic potential of UNBS1450, we attenuated the UNBS1450-induced Na^+^ elicitation by cultivating AML cells in two different isotonic media, containing a physiologic or reduced content of Na^+^ (respectively, 150 and 50 mM). The reduced extracellular Na^+^ concentration protected AML cells from UNBS1450-induced cell death (Fig. 3D–F and Supplementary Fig. S8A). A similar outcome was observed when we supplemented the medium with KCl or added the non-selective K^+^ channel blocker tetraethylammonium (TEA), two strategies preventing the intracellular K^+^ depletion (Fig. 3G–I and Supplementary Fig. S8B, C).

The murine Atp1a1 is more resistant to CG binding due to a residue difference in the N-terminal region [34]. AML cells transfected with a murine *Atp1a1*-expressing plasmid were protected against UNBS1450; this effect was not observed when cells were transfected with the same vector expressing the human ATP1A1 cDNA (Fig. 3J, K).

### BCL2L1 impairs the apoptogenic activity of UNBS1450

**Fig. 4:**
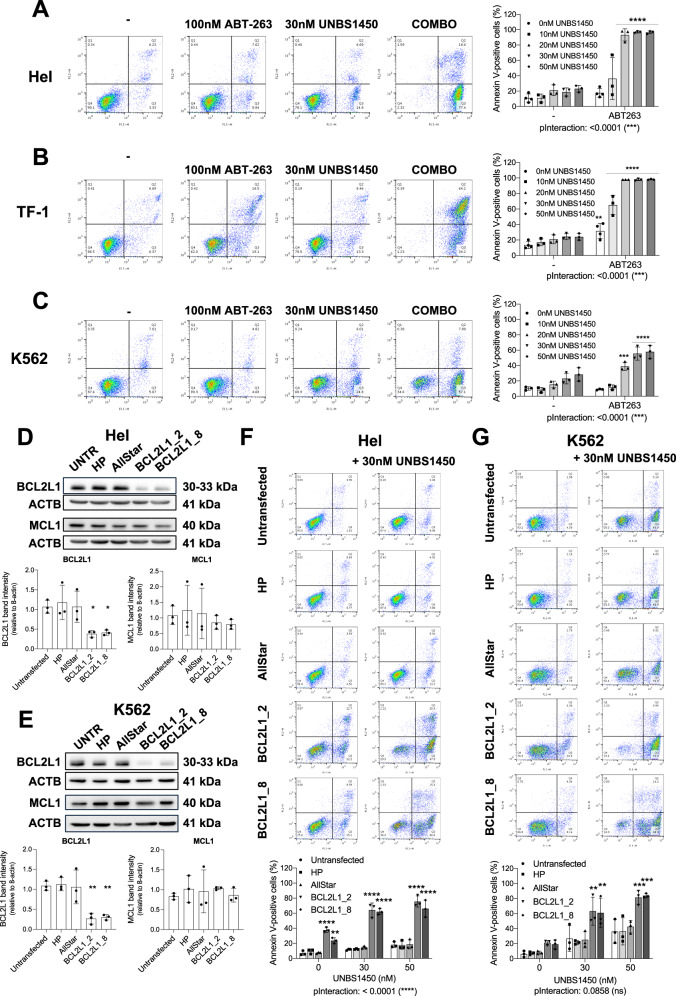
BCL2L1 is a factor of resistance to the CG UNBS1450. Analysis of apoptosis in BCL2L1-overexpressing FAB M6 **A** Hel and **B** TF-1, and **C**. CML K562 cells by Annexin-V/PI viability assay. Cells were treated with 30 nM UNBS1450 (18 h) alone or with 100 nM ABT-263. Genetic *BCL2L1* silencing using 10 nM of two different siRNAs (BCL2L1_2 and BCL2L1_8). In parallel, cells were untransfected; transfected with the transfection buffer only (HP); or 10 nM of a control siRNA (AllStar). **D**, **E** Western blot analyses confirmed BCL2L1 downregulation without MCL1 level modulation in Hel and K562 cells. **F**, **G** Apoptosis induction by UNBS1450 in transfected cells (18 h, 30 nM). *N* = 3. Two-way ANOVA; post-hoc: Sidak; statistical significance: **<0.01, ***<0.001, ****<0.0001.

We expected BCL2L1 to prevent the apoptotic commitment elicited by UNBS1450. The BH3-mimetic navitoclax, which inhibits BCL2L1 along with BCL2 and BCL2L2 (BCL2 like 2; also known as BCLW), potently sensitized the two resistant M6 AML TF-1 and Hel to UNBS1450. This effect was common to the chronic myeloid leukemia K562 cell line, also resistant to UNBS1450 and exhibiting high levels of expression of BCL2L1 (Fig. 4A–C and Supplementary Fig. S9A–C). The genetic *BCL2L1* inhibition by RNA interference sensitized these cell models to UNBS1450, similar to navitoclax. As expected, the selective BCL2 inhibitor VEN could not synergize with UNBS1450 in M6 AML, in line with our previous findings [17] and K562 cells (Fig. 4D–G, Supplementary Figs. S9D, E, S10). Overall, these findings reveal that BCL2L1 prevents the cytocidal potential of UNBS1450.

### UNBS1450 inhibits the expression of short-lived proteins downstream to ionic perturbation via protein synthesis inhibition

We previously described the early downregulation of MCL1 by CGs in different cancer cell models; this modulation occurs at the post-transcriptional level in a caspase-independent manner and contributes to cell death priming. At late times, BCL2 remained largely unaffected or showed only minor changes. A cell-type-specific modulation of BCL2L1, whenever occurring, happened concomitantly or after caspase cleavage/activation [15–17]. As CGs downregulate short-lived proteins and induce alterations in the protein synthesis machinery, [16, 35–38] we hypothesized that UNBS1450 reduced the expression of various short-lived proteins, including MCL1 [16], by inhibiting their protein synthesis and that this phenomenon resulted from ionic perturbation.

**Fig. 5:**
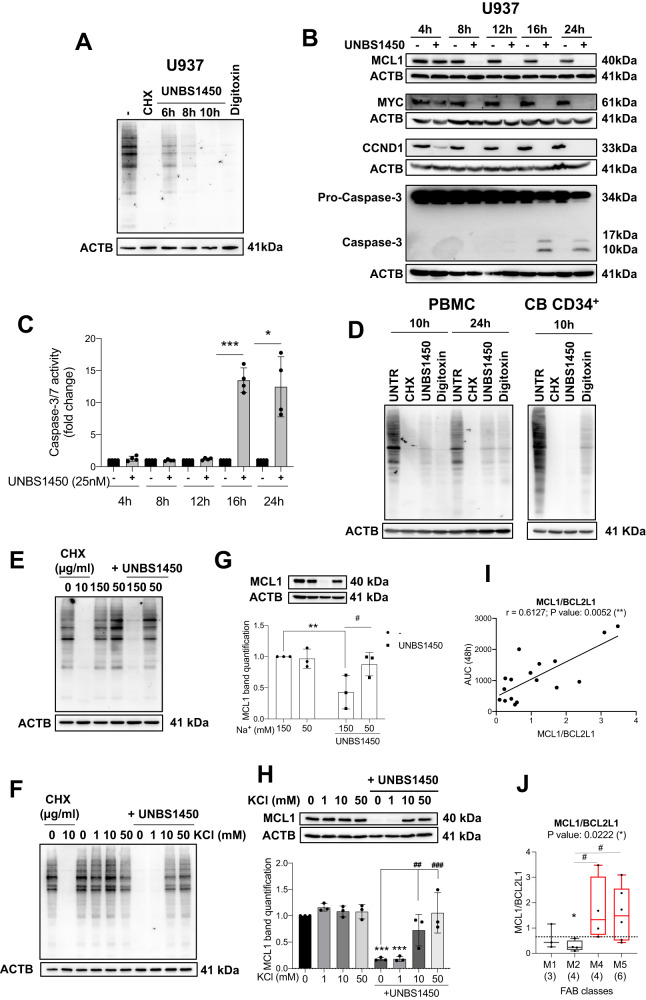
UNBS1450 inhibits the expression of short-lived proteins downstream to the ionic perturbation and via inhibition of protein synthesis. **A** Protein synthesis assay monitored (*N* = 3) and **B** Kinetic western blot analysis of MCL1, MYC, and CCND1 in U937 cells (25 nM UNBS1450), paralleled by the analysis of caspase-3 (CASP-3) cleavage (*N* = 3) and **C** caspase-3/7 activity assay (*N* = 4; Two-way ANOVA; post-hoc: Sidak; *P* values: *<0.05, ***<0.001). Protein synthesis assay in **D** PBMCs, proliferating CB-CD34^+^ (*N* = 4), and in U937 cells upon **E** KCl supplementation or **F** cultivation in salt-balanced modified media containing 150 or 50 mM Na^+^ (*N* = 3). **G** and **H** Western blot analysis of MCL1 expression levels with relative band quantification in the same condition of panels **E** and **F** (N = 3). Statistical significance between UNBS1450-untread and treated cells (*) or between UNBS1450-treated samples without or with KCl supplementation (#): Two-way ANOVA; post-hoc: Sidak; *P* values: */#<0.05, **/##<0.01, ***/###<0.001). **I** Correlation between UNBS1450 AUC values and MCL1/BCL2L1. MCL1 expression values were retrieved from the previous study on the same specimens [17]. **J** MCL1/BCL2L1 ratio according to FAB subtype in AML patient blasts (*N* = 17). Kruskal–Wallis test for median comparison; Mann–Whitney test for comparisons between the median of each subgroup and the overall median (dashed line; *) or between specific subgroups (#); *P* values: */#<0.05).

The Connectivity Map (CMap) dataset (www.clue.io) [39] includes 12 CGs (Supplementary Fig. S11A). The pertubagen type “protein synthesis inhibitor” shows the highest score of signature similarities to this pool of ATP1A1-targeting ATPase inhibitors [35]. Next, we queried CMap using as input the list of genes that were >1.5-fold up-regulated or <0.5-fold down-regulated after nine hours of treatment of the M5 U937 cells with UNBS1450 [16] (Supplementary Tables S13 and 14). CMap assigned the highest score to “protein synthesis inhibitor” after “ATPase inhibitor” perturbagens (Supplementary Fig. S11B). In line with these findings, UNBS1450 inhibited protein synthesis, like the protein synthesis inhibitor cycloheximide (CHX) and the CG digitoxin (Fig. 5A). MCL1 downregulation was concomitant with this modulation. Furthermore, the short-lived proteins MYC (MYC proto-oncogene, bHLH transcription factor) and CCND1 (cyclin D1), two bona fide markers of protein synthesis efficiency/inhibition, were downregulated as MCL1 before caspase activation (Fig. 5B, C and Supplementary Fig. S12A). The inhibitory potential of UNBS1450 on protein synthesis took place in both sensitive and resistant AML cell models (e.g., M5 THP-1 and M6 Hel) as well as in non-cancer cell models, as the quiescent peripheral blood mononuclear cells (PBMCs), the proliferating cord blood-derived (CB) CD34^+^ myeloid cells, and phytohemagglutinin (PHA)/interleukin (IL)-2-stimulated PBMCs (Fig. 5D and Supplementary Fig. S12B, C). The effect was comparable to the clinically used protein synthesis inhibitor Synribo (omacetaxine mepesuccinate, homoharringtonine, HHT) [40] and efficient at lower concentrations than those required to induce cell death in the UNBS1450- and HHT-resistant Hel cell line but sufficient to modulate MCL1 expression (Fig. 5B, D, and Supplementary Fig. S12B). We excluded the mammalian target of rapamycin (mTOR) pathway inhibition or proteasomal activation (Supplementary Fig. S12D, E). Lower extracellular Na^+^ concentrations and K^+^ supplementation inhibited the modulatory effects of UNBS1450 on protein synthesis and MCL1 expression (Fig. 5E–H), indicating that both events happened downstream of the ionic perturbation.

MCL1 downregulation promotes UNBS1450-induced cell death and synergy with VEN in the AML blasts [16, 17]. BCL2L1 is the preferred substitute for MCL1. As we proved that NKA inhibition causes MCL1 downregulation, we assessed whether the MCL1/BCL2L1 ratio predicted the AML blast response to UNBS1450, like the ATP1A1/BCL2L1 ratio. In our AML patient cohort, the MCL1/BCL2L1 ratio was significantly associated with the AML blast sensitivity against UNBS1450 (Rs: 0.6127, *p*-value: 0.0052). FAB M4 and M5 AML patients exhibit the highest MCL1/BCL2L1 ratios (Fig. 5I–J and Supplementary Fig. S12F, G). Further stratification of the AML patients in primitive (M0-M2) vs. differentiated (M4-M5) subgroups did not provide any significance; Additionally, we did not find a significant correlation between the MCL1/BCL2L1 ratio and UNBS1450 sensitivity in our panel of 14 cell lines (Supplementary Fig. S12G, H). MCL1/BCL2L1 showed a similar distribution to ATP1A1/BCL2L1 among FAB subtypes in AML patient (*p*-value < 0.001) and cell line cohorts (*p*-value = 0.0018); Supplementary Fig. S13A–C). *MCL1*/*BCL2L1* was also differentially expressed and higher in the relapsed than the primitive clone present at diagnosis (*p*-value < 0.001 for both Dx-prim vs. RI-mono and Dx-mono vs. RI-mono), differently from relapsed AML samples from conventional chemotherapy (Supplementary Fig. S13D, E). MCL1/BCL2 expression also significantly correlated with UNBS1450 sensitivity and FAB stratification. FAB M4 and M5 showed the highest values (Supplementary Fig. S12F). Restricted analysis of correlation studies to FAB subtypes (M1-M0 vs M4-M5) did not show significance. Furthermore, the correlation between MCL1/BCL2L1 and MCL1/BCL2 ratios and UNBS1450 sensitivity was not consistently significant in the panel of tested cell lines (Supplementary Fig. S12G). These findings suggest that the ATP1A1/BCL2L1 and MCL1/BCL2L1 ratios are mechanistically linked. Overall, we concluded that ATP1A1/BCL2L1 is the ubiquitous predictor of CG response in the investigated in vitro/ex vivo settings.

### The translational potential of CGs

**Fig. 6:**
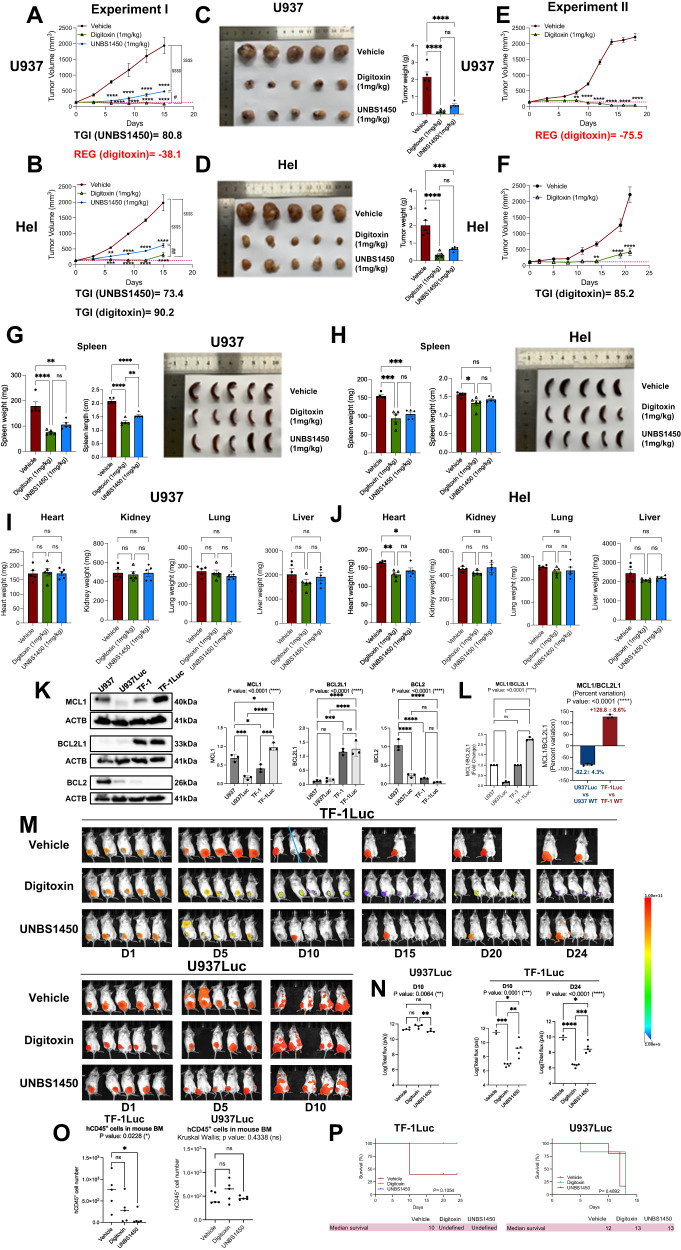
The translational potential of CGs. Tumor volume (mm^3^) changes in subcutaneously injected **A** M5 U937 and **B** M6 Hel xenografts treated with UNBS1450 or digitoxin (1.0 mg/Kg) or vehicle (*N* = 5 mice/group). **C**, **D** Tumor weights assessed in the same mice groups 24 h after the last treatment. **E**, **F** The same analysis as in **A** and **B** in experiment II (U937: *N* = 5 mice/group; Hel: *N* = 4 mice/group). Mouse spleen weight and length of each treatment group (experiment I) in **G** M5 U937 and **H** M6 Hel. Weight of the indicated organs for each treatment group (experiment I): **J** M5 U937 and **K** M6 Hel. Mean ± SEM. Dashed line: average of all the tumor volumes at T0. TGI tumor growth inhibition, REG tumor regression. **K** Western blot analysis and quantification of MCL1, BCL2L1, and BCL2 proteins in U937Luc and TF-1Luc cell lines compared to the parental WT cells used in this study (*N* = 3). **L** Fold change (left) and percent of the fold change variation (right) of MCL1/BCL2L1 protein ratio in luciferase reporter vs. parental cell lines. **M** Bioluminescence imaging and **N** quantification at the indicated times of TF-1Luc and U937Luc xenografts treated with UNBS1450 or digitoxin (0.5 mg/Kg) or vehicle (*N* = 5 mice/group). **O** Flow cytometric quantification of human CD45^+^ cells in the bone marrow from the right femur and tibia, harvested at the end of the experiment on the same mice (number of positive cells/ 10,000 events recorded). **P** Kaplan–Meier survival curves of vehicle vs. CG-treated TF-1Luc or U937Luc xenografted-mice (log-rank (Mantel–Cox) test). Statistical analysis: **A**, **B**, **E**, **F** One-Way ANOVA; post-hoc: Tukey (trend); Dunnett (comparison at the endpoint between groups). **C**, **D**, **G**–**L**, left panel-**N**: One-way ANOVA; post-hoc: Tukey; Sydak (**K**-left panel-**N**). **O** Kruskal–Wallis (post-hoc Dunn’s). **L** (right panel): unpaired T-test (two-tailed). *P* values: */#<0.05, **/##<0.01, ***/<0.001, ****/^$$$$^<0.0001.

Next, we investigated the anti-tumor potential of CGs in vivo. Overall, UNBS1450 and digitoxin reduced the tumor volume and weight in M5 U937 and M6 Hel subcutaneously injected BALB/c nude mice (Fig. 6A–D, and Supplementary Fig. S14A–D). Digitoxin stably impacted tumor growth, evolving into a tumor regression in U937 xenografts (regression (REG): −38.1) at the endpoint; in Hel xenografts, the tumor volume remained unchanged during the early phases of the treatment to eventually increase before the endpoint (Fig. 6B and Supplementary Fig. S14E–G). UNBS1450 induced tumor growth inhibition (TGI); however, the difference between the two xenograft models was more modest. The endpoint histological analysis showed a significant reduction of the Ki-67 proliferation index; in contrast, there was no or only very modest detection of TUNEL-positive cells in both xenografts (Supplementary Fig. S14H, I). These results prevent discerning between an absence of apoptosis induction or early elimination of dying cells. The treatment of an additional group of U937 and Hel xenografts for a longer time, compatible with the analysis in the control group, with digitoxin confirmed a stable tumor regression in U937 xenografts (REG: −75.5). Again, tumor growth started to recover in digitoxin-treated Hel xenografts after the initial phase of stable disease (Fig. 6E, F; Supplementary Fig. S14C, F, G, and Supplementary Table S15). Overall, the treatment was well-tolerated. CGs did not induce any relevant weight loss (below 12% of the relative body weight for all animals) nor generally impacted organs. Spleen size and volume were reduced (Fig. 6G–J, Supplementary Fig. S14J, K, and Supplementary Table S16).

CGs were assessed for their translational potential by intrafemoral injection of AML cell lines into immunodeficient NSG mice. This experimental setting allows bone marrow AML cell engraftment (five mice/group; see Supplementary Fig. S14L for the experimental scheme). We selected the luciferase reporter cell lines U937 (U937Luc) and TF-1 (TF-1Luc; see Supplementary Methods for the cell line source and the experimental procedures) to match the characteristics of the WT cell lines used for the mechanistic investigations. Indeed, the WT TF-1 cells shared similarities with the Hel cells related to protein expression patterns and response to UNBS1450 (see Figs. 2D–F and  4A, B; Supplementary Figs. S9A, B,  S10A, B and S16C–G). Overall, we expected the two luciferase reporter cell models to recapitulate in vivo the differential CG sensitivity observed in other experimental settings with the corresponding WT cell lines. Considering that MCL1 is a fundamental target of CG, downregulated downstream to NKA binding, and MCL1/BCL2L1 ratio is associated with CG sensitivity, we compared the expression pattern of the anti-apoptotic proteins MCL1, BCL2L1, and BCL2 in WT vs. luciferase reporter cells for both U937 and TF-1 cell lines. Importantly, we observed that the luciferase reporter cell lines expressed a different BCL2 family protein repertoire compared to the WT counterparts: In U937Luc cells, MCL1 protein expression was barely detectable. In contrast, TF-1Luc cells showed much higher MCL1 protein expression levels than WT TF-1 and, overall, the highest expression levels compared to WT and U937Luc cells (Fig. 6K). In addition, BCL2 protein expression was reduced in both luciferase reporter cell lines. Whereas WT U937 expressed high levels of BCL2, U937Luc cells were almost devoid (Fig. 6K). No relevant changes were observed for the BCL2L1 protein levels in WT/Luc cell lines. The MCL1/BCL2L1 ratio was consequently strongly reduced in U937Luc compared to WT U937 and increased in TF-1Luc cells with respect to WT TF-1 (Fig. 6L). These findings let us hypothesize an inversed differential CG sensitivity in the two Luc NSG mice xenograft models in correlation with the antipodal MCL1 expression levels observed in both Luc cell models with respect to the WT cell lines. It has not been reported before that AML Luc and WT cell lines exhibit such opposite expression patterns of BCL2 family proteins, which highlights the need for caution when studying apoptogenic mechanisms. In line with our hypothesis, in vivo imaging analysis showed that digitoxin and UNBS1450 significantly reduced AML cell burden compared to the vehicle-treated mice in TF-1Luc xenografts. CG treatments did not prevent the disease progression and the pronounced ability of U937Luc cells to disseminate (Fig. 6M, N and Supplementary Fig. S14M). The modulatory effect was observed in TF-1Luc xenografts treated with CGs until the animals were sacrificed at day 24, 9 days after the end of treatment (Fig. 6N, right panel, and Supplementary Fig. S14M, right panel). In line with these results, the number of human CD45^+^ blasts in the bone marrow of TF-1Luc xenografts was reduced (significant with UNBS1450 treatment; Fig. 6O). Furthermore, all U937Luc xenografts succumbed shortly independently of the treatment received (median survival: 12 days in vehicle-treated mice vs. 13 days in CG-treated mice). After 24 days, all TF-1Luc mice treated with digitoxin or UNBS1450 remained alive compared to controls. This made the median survival time undefined in CG-treated mice vs. the 10 days estimated for vehicle-treated mice, although the Kaplan–Meier analysis did not reach the significance (Fig. 6P). In agreement with our general hypothesis, U937Luc^MCL1/BCL2L1_LOW^ xenografts were resistant to CG treatment, while TF-1Luc^MCL1/BCL2L1_HIGH^ xenografts were sensitive.

Next, we explored combination regimens that may benefit from including CGs. The CG UNBS1450 and VEN synergize across multiple experimental models [17]. Our rationale for this combination was targeting MCL1 with CGs [41]. Here, we explored the association between cell type and drug resistance. The ex vivo AML drug resistance study from the BeatAML2 cohort [23] confirms VEN resistance as strongly associated with the monocyte-like cell phenotype. In this dataset, ex vivo VEN response (AUC values) were significantly anti-correlated with *ATP1A1*/*BCL2L1* and *MCL1*/*BCL2L1* expression (Supplementary Fig. S15A, B). The monocyte-like cell type also correlates with resistance to AZA (Supplementary Fig. S15A) [23], that is administrated with VEN to intensive chemotherapy-unfit AML patients. Combining UNBS1450 or digitoxin with the HMAs AZA or decitabine (DAC) was additive to synergistic in a panel of M4/M5 AML cell lines. No significant correlation between AZA drug response and the combined markers of interest emerged from the BeatAML2 cohort (Supplementary Fig. S15C, D).

Altogether, our studies document that CGs impact AML tumor growth and are well-tolerated in vivo. Digitoxin induced tumor regression in M5 AML U937 xenografts and tumor growth inhibition in M6 AML xenografts. The combination with VEN and HMAs could represent important future therapeutic areas to explore.

## Discussion

The multifactorial marker *ATP1A1*/*BCL2L1* improves the prediction of the response of cancer cells to CGs. We suggest AML patients bearing intratumoral myelomonocytic and monocytic (FAB M4/M5) subclones as suitable recipients of CG-based regimens. Besides, patients with *CBFB* core and *KMT2A* rearrangements or *FLT3* missense mutations are possible interesting candidates.

Despite their known pharmacokinetics, CGs have not yet met the criteria for repositioning in cancer therapy. The validation of the biological target(s) and subsequent bona fide markers of response remains a major conundrum. Diving into the registered clinical trials involving CGs (alone or combined with approved therapeutic drugs), the enrolled patients are frequently affected by solid tumors, including recurrent and metastatic prostate, pancreatic, lung, and breast cancer. The same phase I trial with UNBS1450 was on patients with solid tumors or lymphomas. Several solid tumors, like sarcomas, prostate, breast, pancreatic, lung, colorectal, and liver carcinomas, show *ATP1A1*/*BCL2L1* levels below the average in the TCGA pan-cancer analysis. CML and B-cell lymphomas also belong to the low-expressing group (Fig. 2H). These tumors might exhibit underestimated intrinsic resistance to CGs, which our combined marker more efficiently intercepts. Alternatively, potentially targetable cancer types emerge (i.e., Ewing’s sarcoma and melanoma showing the highest *ATP1A1*/*BCL2L1* levels). These findings imply that the *ATP1A1*/*BCL2L1* ratio is also associated with non-AML cancer types and should be considered as an independent predictor of CG response.

The AML differentiation state affects the success of current therapeutic combinatorial regimens. Specifically, myelomonocytic and monocytic AML blasts are resistant to VEN-based regimens [1, 4, 5]. Here, we provided evidence that the *ATP1A1*/*BCL2L1*^high^ phenotype is a distinctive clinical feature of these AML subtypes, preserved in VEN-relapsed patients [1] and consistently associated with VEN resistance in the ex vivo drug response [23]. Recently, there has been doubt about using the monocytic maturation stage as a predictor of VEN-based regimens when translating ex vivo results into clinical settings. The flow cytometric quantification of CD64^+^/CD11b^+^ (ITGAM) blasts and monocyte counts were inconclusive [8, 9]. Notably, a very recent study postulates the existence of monocytic LSCs, developmentally and phenotypically distinct from primitive LSCs, which would be responsible for monocytic AML progression after VEN-based therapy. This novel LSC type would rely more on MCL1 to support metabolism than BCL2 [10]. These observations suggest that selected monocytic-like phenotypes may contribute to refractoriness/relapse to VEN. These LSCs, destined to develop monocytic AML, could so far not yet be identified with adequate biomarkers.

*ATP1A1*/*BCL2L1* was lower expressed in healthy mononuclear cells and monocytes from donors (Fig. 1 and Supplementary Fig. S3). Further investigations are required to confirm that a higher ATP1A1/BCL2L1 ratio is distinctive of monocytic AML. We can make some considerations, nevertheless. CGs were not toxic to monocytes from healthy donors (Supplementary Fig. S7); moreover, the clinical use of CGs did not lead to monocytopenia. Monocyte-derived macrophages are more sensitive than non-adherent peripheral blood mononuclear cells to the cytocidal activity of the CG ouabain [42]; this vulnerability may be exploited to target the pathogenic macrophage infiltration in the white adipose tissue occurring in obesity and metabolic syndromes [43]. It will be interesting to verify whether the expression of (or dependency from?) higher *ATP1A1*/*BCL2L1* levels might be a prominent vulnerability of diseased monocytes and monocyte-derived cells. Of note, further stratification of AML patients in primitive (M0-M2) vs. committed subtypes (M4-M5) potentiates the predictive power of the ATP1A1/BCL2L1 ratio (Fig. 2C). Although these findings require further consolidation on larger patient cohorts, they confirm a homogeneous higher expression level of the ATP1A1/BCL2L1 ratio in mature AML subtypes, more vulnerable to CGs.

CGs inhibit MCL1 protein expression [15–17]. Both MCL1/BCL2L1 and MCL1/BCL2 ratios significantly correlate with UNBS1450 sensitivity in our AML cohort; furthermore, they significantly correlate with FAB stratification, with FAB M4/M5 showing the highest values. This result aligns with the higher expression of MCL1 and lower expression of BCL2 in monocytic compared to primitive AML blasts [5]. We previously found that BCL2 expression is not or barely impacted by UNBS1450 at late times or doses higher than those required to induce cell death commitment. Furthermore, BCL2 overexpression did not rescue BCL2/MCL1 co-dependent U937 cells from UNBS1450 [15, 16]. In contrast, the same cell model was fully committed to apoptotic cell death when UNBS1450 was combined with BCL2 inhibitors (the selective venetoclax or the broader navitoclax). Notably, this effect was achieved with sub-apoptogenic concentrations of UNBS1450 impacting MCL1, not BCL2L1 and BCL2 [17]. We previously documented that the decrease in BCL2L1 expression depends on the cell context and does not occur in UNBS1450-resistant cells [16], which can be re-sensitized by chemical or genetic BCL2L1 inhibition (Fig. 4). BCL2L1 and MCL1 have redundant biological functions whenever MCL1 is targeted [41, 44]. Altogether, these results suggest that BCL2 and BCL2L1 are not equivalent in the mechanism of action of CGs. BCL2 modulation is dispensable, while BCL2L1 modulation might be essential to replace early downregulated MCL1. Notably, the ATP1A1/BCL2L1 but not the ATP1A1/BCL2 ratio significantly correlates with UNBS1450 sensitivity (in vitro and ex vivo settings), supporting a more potent role of BCL2L1 than BCL2 as a resistance factor to CGs. However, cancer cells relying on BCL2 and lacking MCL1/BCL2L1 might also be more resistant to CGs. Overall, the modulation of the BCL2 protein expression downstream to the ionic perturbation emerges as a crucial determinant for the anti-cancer effects of CGs as a single agent and even more in combination with BCL2 inhibitors.

**Fig. 7:**
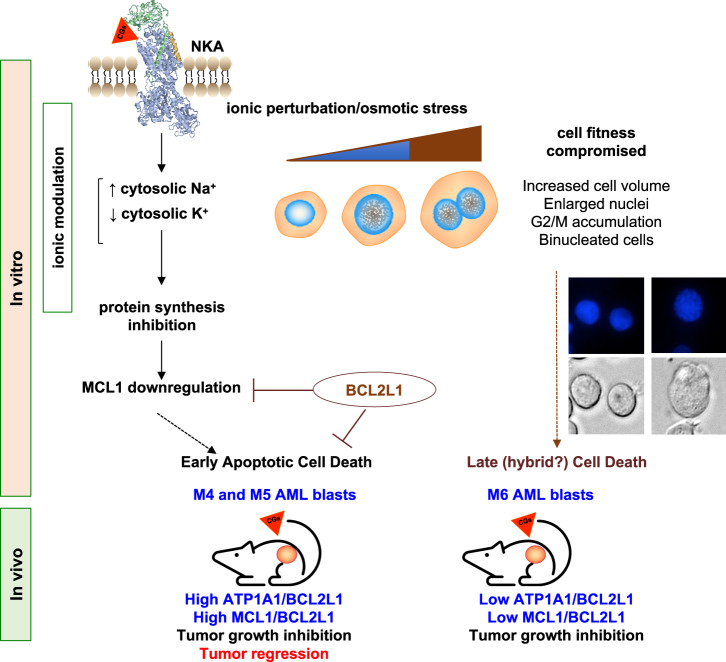
A mechanistic model for the action of UNBS1450. The 3D NKA image is from FirstGlance, Jmol; https://www.bioinformatics.org/firstglance/fgij/fg.htm?mol=3kdp.

Our findings indicate that the MCL1 downregulation depends on NKA inhibition. The ionic perturbation may likely activate a fatal osmotic stress response (Fig. 7). Cell types expressing low BCL2L1 levels (i.e., myelomonocytic M4 and monocytic M5 AML) would be early committed to apoptosis; cells expressing high BCL2L1 levels would bypass this commitment. However, they manifest cell cycle progression alterations leading to a G2/M arrest (Supplementary Fig. S16). This outcome occurred in BCL2L1-overexpressing cells, independently of the tissue origin and CG used [45].

We did not detect TUNEL-positive cells at the endpoint of CG treatment in vivo. This result prevents us from concluding whether dying cells were progressively eliminated or CGs triggered a strong tumor growth inhibition in animal models. However, we only observed net tumor regression in M5 U937 xenografts; in the M6 Hel model, the tumor growth resumed. Two independent sets of AML xenografts subcutaneously injected confirmed this result with the CG digitoxin (Fig. 6 and Supplementary Fig. S14H, I). We extended our translational studies to NSG mice intrafemorally injected with luciferase reporter AML cell lines, a model allowing bone marrow engraftment of AML cells. Remarkably, we found that Luc cell models exhibit a profound alteration of the anti-apoptotic BCL2 protein expression pattern. U937Luc cells were substantially devoid of MCL1, the essential target of CGs together with the NKA. Conversely, TF-1Luc cells expressed much higher levels of MCL1 than the WT cell line and the highest MCL1 levels when comparing U937 and TF-1, WT, and Luc cells (Fig. 6K). The altered MCL1 expression pattern let us hypothesize that the two Luc cell xenograft models could corroborate our mechanistic model. However, we anticipated that they would not recapitulate the differential CG sensitivity observed with the corresponding WT cell lines. Indeed, U937Luc cells were strongly refractory to CGs (both digitoxin and UNBS1450), as substantially devoid of MCL1. In contrast, CGs significantly reduced TF-1Luc cell survival and proliferation. Bioimaging studies, quantification of human CD45^+^ cells in the bone marrow, and survival analysis provided consistent results (Fig. 6M–P). As TF-1Luc cells express high levels of MCL1, even higher than WT U937 cells, they may have developed a stronger MCL1 reliance and are more sensitive to CG treatment than U937Luc cells used here for comparative studies. Consequently, the MCL1/BCL2L1 ratio is also affected: it is drastically reduced in U937Luc and increased in TF-1Luc cells (-82% and +125% with respect to the WT cell lines).

Luc cells are frequently used for testing in vivo combinatorial regimens, including inhibitors of BCL2 family proteins. Generally, no comparative analysis of BCL2 protein expression between the WT and Luc-modified cell models is shown, assuming that Luc cells maintain the same BCL2 expression protein and dependency patterns as the WT cell models. We found additionally that BCL2 protein expression was generally reduced in Luc cell models (high BCL2-expressing WT U937 and very low BCL2-expressing U937Luc cells). No changes in BCL2L1 expression were observed. These results let us suspect a more extensive change in the BCL2 family expression pattern and, potentially, a diversified BCL2 protein reliance in Luc-modified cells. A global BCL2 profiling will be required to identify cell-specific BCL2 dependency. Furthermore, we cannot exclude additional alterations in global protein expression, potentially involving other pro-survival factors or mediators essential for the CG mechanisms. Our comparative analysis warns about the risk of a so far underestimated BCL2 protein expression reprogramming in Luc-expressing cell models. In this sense, the Luc-modified AML cell models become suitable cellular tools to track drug efficacy in vivo in a non-invasive way; their response to treatment, however, may be potentially unrelated and no longer representative of their parental cell lines. Overall, we cannot exclude that a further optimization of CG concentrations might be required in in vivo settings. For example, pharmacokinetics-related factors, like CG drug stability and clearance, remain to be further investigated.

Also, *MCL1*/*BCL2L1* expression significantly correlates with ex vivo VEN resistance (Supplementary Fig. S15). UNBS1450 and VEN synergized on AML patient blasts and cell lines exhibiting a BCL2/MCL1 co-expression pattern [17]. Our explorative screening indicates effects ranging from additive to synergistic effects of CGs/HMAs combinations. These observations inspire the future assessment of the efficacy of CG/VEN/HMAs triplets. An off-label clinical trial involving clinically approved CGs might be considered on de novo/relapsed AML patients eligible for VEN-based regimens. The myelomonocytic/monocytic phenotype of AML blasts would be an important inclusion criterion when enrolling patients and validating the *ATP1A1*/*BCL2L1* and *MCL1*/*BCL2L1* expression as response markers. Translation to therapy will require the evaluation of potential drug interactions. VEN is a P-glycoprotein (P-gP) inhibitor; the CG digoxin is a P-gP substrate [46]. Interactions between P-gP and digitalis-like compounds are known [47]. The co-administration of a single oral dose of digoxin (0.5 mg) and VEN (100 mg) increased the maximal plasma concentration of digoxin in healthy female volunteers [46]. A combination between VEN and CGs might require specific administration schedules to minimize drug-drug interaction and avoid potentiating the risk of toxicity associated with these drugs [46, 48]. It will be important for the optimal patient stratification to establish whether the detection of ATP1A1/BCL2L1 at mRNA or protein level might be equivalent. An excellent correlation between mRNA and protein levels for BCL2L1 (Pearson: 0.831; Spearman: 0.754) and an mRNA/protein correlation in the instance of ATP1A1 in the averaged values (Pearson: 0.493; Spearman: 0.545) emerge from the CCLE quantitative proteomics of 375 cell models of different origins [49]. We observed similar results when restricting this analysis to a list of blood cancer cell lines included in the Depmap portal (Supplementary Fig. S17). These data might be informative of the most suitable expression level to select by clinicians for patient stratification. Specific mutations affect the correlation between drug response and cell type score [23]. It will be important to assess potential co-variates influencing drug sensitivity prediction to improve the robustness of the combined markers suggested here. We found a significant additional association between specific cytogenetic alterations (e.g., *CBFB* and *KTM2A* rearrangements) and FLT3 missense mutations. CBFB core results are enriched in FAB M4 from adult TCGA and pediatric TARGET AML, whereas the pediatric *KMT2A* rearrangements are enriched in FAB M5 patients. *FLT3* KTD is enriched in FAB M4/M5 AML patients (Supplementary Fig. S4). These associations may therefore be the consequence of the myelomonocytic/monocytic-like phenotypes.

In conclusion, this study provides preclinical evidence of markers improving the prediction of AML patient response to CGs. In the future, biomarker combinations integrating CG response mechanisms will allow for elaborating a multifactorial score to stratify patients more efficiently. As CGs are clinically used compounds with well-known side effects and pharmacokinetics, our findings provide detailed insight for repurposing CGs. AML patients with intratumoral myelomonocytic and monocytic subclones might be the most suitable candidates for translational investigations.

### Supplementary information

Supp mat

Supp tables

## Data Availability

Patient-related clinical data are available in a previously published study [17]. RNA-seq and CITE-seq data come from publicly available cohorts (Supplementary Data). The transcriptomic data on CG-treated U937 cells are deposited in GEO (GSE61956) and belong to a previously published study [16]. The data supporting the findings of this study are available from the corresponding author upon request.
