# Supplementary material for: ATP1A1/BCL2L1 predicts the response of myelomonocytic and monocytic acute myeloid leukemia to cardiac glycosides
Source: Leukemia. 2023 Oct 30;38(1):67–81. doi: 10.1038/s41375-023-02076-8 (PMC10776384; doi:10.1038/s41375-023-02076-8)
Supplement: Supplementary file 1 — Supp mat [file 41375_2023_2076_MOESM1_ESM.pdf]

ATP1A1/BCL2L1 predicts the response of myelomonocytic and monocytic acute myeloid leukemia to cardiac glycosides

Claudia Cerella, Sruthi Reddy Gajulapalli, Anne Lorant, Deborah Gerard, Florian Muller, Yejin Lee, Kyung Rok Kim, Byung Woo Han, Christo Christov, Christian Récher, Jean-Emmanuel Sarry, Mario Dicato, and Marc Diederich

Supplementary Methods

Ethical aspects and isolation/cultivation of primary cells

Stored frozen samples from AML patients were obtained from the HIMIP collection (BB-0033-00060). According to French law, HIMIP collection has been declared to the Ministry of Higher Education and Research (DC 2008-307 collection 1) and obtained a transfer agreement (AC 2008-129) after approbation by the "Comité de Protection des Personnes Sud-Ouest et Outremer II" (ethical committee). Clinical and biological annotations of the samples have been declared to the CNIL (Comité National Informatique et Libertés (Data processing and Liberties National Committee); samples were obtained from patients diagnosed with AML at the Toulouse University Hospital (TUH) after signed informed consent per the Declaration of Helsinki. AML specimens with viability < 70% (trypan blue assay) at 0 hours of treatment were excluded from further analysis (N=23). Healthy CB was kindly donated by Clinique Bohler (Luxembourg, Luxembourg) after written informed consent and in agreement with the National Committee of Research Ethics in Luxembourg (Luxembourg). PBMCs were obtained from Red Cross Luxembourg (Luxembourg, Luxembourg) after the donor's informed written consent and Red Cross ethical approval (LBMCC-2019-0001; LBMCC-2019-0002). After isolation, a fraction of PBMCs was incubated with a cocktail of mitogens/cytokines, including phytohemagglutinin (PHA; Gentaur, Kampenhout, Belgium) and interleukin-2 (IL-2; Roche, Luxembourg, Luxembourg) to stimulate PBMC proliferation (pPBMCs). The

extraction/isolation and quality control protocols have been previously detailed [1].

Cell lines, culture conditions and cell authentication

Human AML cell lines Hel (ACC 11), MOLM-13 (ACC 554), MV4-11 (ACC 102), OCI-AML3 (ACC 582), TF-1 (ACC 16), THP-1 (ACC 16), U937 (ACC 5), the chronic myeloid leukemia K562 cells (ACC 10), and the non-small cell lung cancer A549 (ACC 107) cell line were from the Deutsche Sammlung von Mikroorganismen und Zellkulturen (DSMZ, Braunschweig, Germany); the peripheral blood B lymphocytes RPMI-1788 (CCL-156) and the neuroblastoma SH-SY5Y (CRL-2266) were from the American Type Culture Collection (ATCC; Manassas, VA, USA). U937 luciferase cells (U937Luc) were a gift from Dr. Jean-Emmanuel Sarry's lab. TF-1Luc cells (TF-1-Luc2, CRL-2003-LUC2™) were purchased from ATCC in 2023.

OCI-AML3 cells were cultivated in an alpha-MEM medium supplemented with 15% (v/v) fetal calf serum (FCS; Lonza). RPMI-1788 cells were cultivated in RPMI 1640 (Lonza, Verviers, Belgium) supplemented with 20% (v/v) fetal calf serum (FCS; Lonza). TF-1 cells were cultivated in RPMI 1640 supplemented with 10% (v/v) fetal calf serum (FCS; Lonza) and 5ng/ml of recombinant human granulocyte-macrophage colony-stimulating factor (GM-CSF; Reliatech). TF-1Luc cells were cultured in complete growth media containing RPMI-1640 medium (ATCC) supplemented with 10% (v/v) FCS, 1% (v/v) penicillin/streptomycin, 2ng/ml recombinant human GM-CSF and 8µg/ml blasticidin (Biomax, Gyeonggi, Guri City, South Korea). All the other cell models were cultured in RPMI 1640 medium supplemented with 10% (v/v) fetal calf serum (FCS; Lonza). All cells were cultivated in the presence of 1% (v/v) antibiotics/antimycotics (Lonza) in a humidified atmosphere at 37°C and 5% CO_2. We renewed the medium every two or three days and maintained the cells in exponential growth, according to the provider's indications. Cells were bi-monthly checked for Mycoplasma contamination

(MycoAlert™ Mycoplasma detection kit, Lonza). Cell line authentication and purity were confirmed, respectively, by DNA profiling using 17 different and highly polymorphic STR (Short Tandem Repeat) loci and by testing the presence of mitochondrial DNA sequences from rodent cells, including mouse, rat, Chinese, and Syrian hamster species (DSMZ; October 2020, reports available on request).

Treatments conditions

AML cell lines and RPMI-1788 were seeded at a concentration of 3.0 x 10^5 cells/ml in a prewarmed complete medium one h before any treatment. After isolation, PBMCs were incubated overnight at a concentration of 2.0 x 10^6/ml in a humidified atmosphere at 37°C and 5% CO_2; the day after, their concentration was adjusted to 1.0 x 10^6/ml in a prewarmed complete medium and incubated one h before any treatments. After 72h of incubation with mitogenic/blastogenic stimuli, pPBMCs, and CB $CD34^+$ concentrations were estimated, and quality controls were performed. Then, cells were seeded at a concentration of, respectively, 1.0 x 10^6/ml and 3.0 x 10^5 cells/ml in a prewarmed complete medium and treated for one h after seeding.[1,2] Adherent cell models were seeded at a concentration of 3.0 x 10^5 cells/well (6-well plate; 2ml of medium); after 24h cells, the medium was replaced, and treatment was added one h later.[3,4]

Analysis and quantification of cell death

Cell death was analyzed and quantified by the following approaches. *i*) Estimation of the percentage of cells with apoptotic fragmented/condensed nuclei (1µg/ml Hoechst, Sigma-Aldrich, Bornem, Belgium; 20min incubation at 37°C) by fluorescence microscopy (Olympus, Hamburg, Germany).[2] The images were analyzed/elaborated using Cell^M software (Olympus Soft Images Solutions GmbH, Germany). *ii*) Mitochondrial membrane potential (MMP) loss

(100nM MitoTracker® Red (MTR) CMXRos, Molecular Probes, Invitrogen, Fisher Scientific, Tournai, Belgium; 30min of incubation at 37°C); *iii*) phosphatidylserine exposure/plasma membrane permeabilization (FITC or APC Annexin V Apoptosis Detection kit; BD Pharmingen, Erembodegem, Belgium) according to manufacturer's instructions. Values (10,000 events) for MTR and Annexin V/PI (propidium iodide) staining were monitored and recorded at the FACS (FACSCalibur, Becton Dickinson, San José CA, USA).[2] Enzymatic caspase 3/7 activity was determined using Caspase-Glo ® 3/7 Assay; Promega, Leiden, The Netherlands). At the indicated time points, 50µl of cell culture were mixed with an equal volume of the reconstituted buffer according to the manufacturer's instructions and incubated on a shaking platform for one h before luminescence detection (reading: 1s; Orion Microplate Luminometer (Berthold, Pforzheim, Germany). Values are reported as relative luminescence units (RLU) or fold-change compared to untreated cells (when multiple controls were required, i.e., kinetic analysis or cells transfected with different constructs). A multi-parametric approach was applied using the abovementioned methods to compare *in vitro* UNBS1450 cytotoxicity. IC50 values were calculated with Compusyn software[5] (https://www.combosyn.com) after selecting one of the three methods providing the best cell death assessment/quantification depending on the cell context. For *ex vivo* AML blasts, due to the limited number of concentrations tested (0, 30, and 50nM), the area under the dose-response data generated by graphing the percentage of Annexin-V-positive blasts after 48h UNBS1450 treatment[2] was computed and selected for comparing UNBS1450 cytotoxicity among different AML specimens (herein referred as the area under the curve, AUC). The percentage of annexin-positive blasts in the untreated (0nM UNBS1450) AML blasts was the baseline for estimating the total area. The conventional SCC vs. CD45 gating served to define the AML blast population prior to cell death quantification.

RNA extraction and Real-Time PCR analysis

Total RNA was extracted using the RNA isolation kit (Macherey-Nagel GmbH & Co. KG, Düren, Germany) according to the manufacturer’s instructions. cDNA was synthesized from 0.5μg RNA using SuperScriptTM III first-strand synthesis system and random hexamer primers (SuperScript First-Strand Synthesis System, Invitrogen, Thermo Fisher Scientific). Real-time PCR analysis was performed using the SYBR® Green PCR Master Mix (Power SYBR Green PCR Master mix 1X, Applied Biosystems, Halle, Belgium) according to the manufacturer’s protocol in a 7300 Real-Time PCR System (Applied Biosystem, Lennik, Belgium). Each quantification was performed at least in duplicate. *ACTB* was used for normalization. Human *ATP1A1* primers (For: 5’-CTA- CCT-GGC-TTG-CTC-TGT-CC-3’; Rev: 5’ -GCT-GAC-TCA-GAG-GCA-TCT-CC-3’); *ACTB* (For: 5’-CTCTTCCAGCCTTCCTTCCT-3’; Rev: 5’-AGCACTGTGTTGGCGTACAG-3’). Murine *Atp1a1* primers are: For: 5’-GGACGCTTTTCAGAACGCCTA-3’; Rev: 5’-CCTTCGGGAAACTGTTCGTCA-3’. Oligonucleotides were purchased from Eurogentec (Liege, Belgium).

Immunoblotting

Protein extraction and western blot procedures have been previously detailed [6]. 40μg of proteins (20μg for protein synthesis assay) were loaded. The polyvinylidene difluoride membrane (PVDF; GE Healthcare, Roosendaal, The Netherlands) was incubated with the following primary antibodies. From Sigma-Aldrich, anti-ACTB (1/10000 1h RT 5% milk-PBST; cod. A5441); anti-ATP1A1 (Merck-Millipore, cod. 06-520): 1/1000, ON +4 °C, 5% milk-PBST. From Thermo Fisher Scientific, anti-ATP1A3 (XVIF9-G10; cod. MA3-915) and anti-ATP1B1 (M17-P5-F11; cod. MA3-930): 1/1000, ON +4 °C, 5% milk-PBST (phosphate buffer saline-0.1% Tween, Sigma-Aldrich). From Santa Cruz Biotechnology (Boechout,

Belgium), anti-CASP3 (1/1000, ON +4 °C, 5% milk-PBST; cod. sc-56053). From Cell Signaling Technology (Leiden, Netherlands), anti-EGFR receptor (cod. 4267S), anti-MCL1 (cod. 4572S), anti-RPS6KB1 (ribosomal protein S6 kinase B1; p-P70, cod-. 9234; total, 2708), anti-EIF4EBP1 (eukaryotic translation initiation factor 4E binding protein 1; T70, cod.9455; T37/46, cod. 2855, S65, cod. 13443; total, cod. 9644): 1/1000, ON +4 °C, 5% bovine serum albumin (BSA)-PBST. Anti-BCL2 (1/2000, ON +4 °C, 5% milk-PBST, Calbiochem, Leuven, Belgium; cod. OP60); anti-BCL2L1 (1/1000, ON +4 °C, 5% milk-PBST, BD Transduction Laboratories, Erembodegem, Belgium; cod. 610212); anti-MYC (1/200, ON +4 °C, 5% milk-PBST, BD Pharmingen; cod.51-1485GR). The bands were revealed by chemiluminescence using an ECL system and a LasMini (GE Healthcare; Roosendaal, The Netherlands) and quantified with Image J software (US National Institute of Health, Bethesda, MD, USA). ACTB served as the loading control.

Ion modulation and measurements

To study the role of extracellular Na^+ influx, cells were cultivated one h before treatments in prewarmed balanced salt solutions containing 150mM or 50mM Na^+. 100mM choline chloride (Sigma-Aldrich) replaced NaCl in the Na^+-low medium.[7] The 150mM or 50mM Na^+ media contained all the other components at the same concentrations (5mM KCl, 1mM $MgCl_2$, 2mM $CaCl_2$, 10mM D-glucose (Sigma-Aldrich), 10mM HEPES (MP Biomedicals; Illkirch Cedex, France) 10% FCS, 1% antibiotics/antimitotic, 1% glutamine, 1% pyruvate, 1% non-essential amino acids (Gibco); pH=7.4). The intracellular Na^+ levels were monitored with SBFI, AM, cell-permeant (Invitrogen, Thermo Fisher Scientific, Tournai, Belgium). Cells were washed and resuspended in prewarmed modified Hanks' Salt Balanced Solution (HBSS) + 1% (v/v) FCS at the end of the treatment at the concentration of 3 x 10^5/ml, and treatments were re-added. 1ml of the sample was incubated with 5µM SBFI in a humidified

atmosphere at 37°C/5% CO_2 incubator; 1 ml was without SFBI (unstained sample). After one h, cells were washed, and each sample was resuspended in 200µl of prewarmed modified HBSS + 1% FCS in the presence of the treatments. 100µl were loaded onto a clear bottom, black 96-well plate, and fluorescence was analyzed with a fluorometer tuned at 37°C (SPECTRAMax Gemini EM, Molecular Devices, Thermo Fischer Scientific). Data (endpoints) were recorded and reported as F340/F380 ratio (a.u.). F340/F380 ratios were normalized and expressed as fold-change compared to the values of untreated cells.

To study the role of K^+ depletion, the cell culture medium was supplemented with 0, 1, 10mM, or 50mM KCl; alternatively, with 50mM of the non-selective K^+ channels blocker tetraethylammonium (TEA, Sigma-Aldrich), one h before UNBS1450 treatment.

Functional studies

Gene silencing. Hel and K562 cells were cultured at 3 x 10^5/ml. The day after, 4.8 x 10^5 cells were resuspended in 400µl of complete culture medium and transferred to a 12-well plate equilibrated in the incubator for one hour. After this time, 400µl RPMI 1640 containing 10nM siRNA and 6µl of HiPerFect Transfection Reagent (Qiagen) were added dropwise onto the plated cells. After gently shaking, the plate was placed back into the incubator. After six h, 1,600µl of prewarmed RPMI 1640 medium containing 12.5% (v/v) FCS and 1% antibiotics/antimycotics was added to each well. The plate was incubated for 48h in a humidified atmosphere at 37°C/5% CO_2 incubator before any other handling. In parallel, the modulation of UNBS1450 in high BCL2L1-expressing cells was tested by chemical inhibition, using the BCL2 family inhibitors ABT-199 (venetoclax; BCL2-selective) and ABT-263 (navitoclax; BCL2, BCL2L1, and BCLW) both purchased from Selleck Chemicals (Hussein, The Netherlands).

Murine *Atp1a1* overexpression. According to manufacturer instructions, 2µg of

construct were transfected into 4 x 10^6 Hel cells using an Amaxa Cell Line Nucleofector® Kit V (Lonza). Briefly, cells were cultured at 2 x 10^5/ml two days before transfection. A 12-well plate containing 1.5ml of complete medium was equilibrated for one h in a humidified atmosphere at 37°C /5% CO_2 incubator on the day of transfection. After counting, 4 x 10^6 Hel cells were centrifuged for 10min at 200g, the supernatant was removed entirely, and the pellets were resuspended in 100µl room temperature (RT) reconstituted Nucleofector® Solution containing the plasmid of interest. Alternatively, 2µg of the pmaxGFP® Vector (Lonza) was used to control transfection efficiency. The entire volume was transferred into a provided cuvette and electroporated (program X-005; Nucleofector® 2b device, Lonza). 500µl of pre-equilibrated complete medium was added to the cuvette, and the volume was gently transferred into one well of the 12-well plate. Transfected cells were incubated for 24/48h before any other handling.

***In vivo* studies**

Subcutaneous injection murine model

Six-week-old female athymic BALB/c nude mice were purchased from the Joongah Bio Company, Seoul, South Korea. In brief, U937 and Hel cells were collected from the cell cultures and washed once with PBS. 2x10^6 U937 or Hel cells were subcutaneously injected into the right flanks of the mice. When tumors had a volume of 100mm^3, mice were divided randomly into three groups and treated as follows: a) vehicle, b) UNBS1450 (1mg/kg), and c) digitoxin (Sigma, St Louis, USA, Catalog D5878; 1mg/kg) treatment group. UNBS1450 and digitoxin were dissolved in 0.9% NaCl in water containing 10% DMSO (Sigma, St Louis, USA, Catalog D2650). 10% DMSO was also added to the saline solution for the control group (vehicle). Treatment solutions were dissolved immediately before use and administered intraperitoneal injections every three days for a total of 6 times. Tumor volume was estimated

every three days using a vernier caliper SD500-150PRO digital caliper (Sincon, South Korea) according to the formula: V= (π/6 x Width x Width x Length) mm^3. The body weights were monitored every three days. All mice were sacrificed 24h after the last treatment.

The percentage of tumor growth inhibition (TGI) was estimated according to the formula: %TGI= 100-%Gr (growth) where Gr= 100x(ΔT/ΔC), ΔT = average of the treated tumor volume at time T – average of the treated tumor volume at time T0; ΔC= average of the untreated tumor volume at time T – average of the untreated tumor volume at time T0. The percentage of regression (REG) was calculated when the tumor volume decreased in treated samples with respect to the treatment start (ΔT=T-T0 < 0) as follows: REG= 100 x (ΔT/T0).[8-10] The relative tumor volume changes in Supplementary Fig. S14E-F were calculated for every mouse applying the formula reported above for REG. The relative body weights were estimated as B/B0 for each mouse, where B= body weight in grams at time T, B0= body weight in grams at time T0.[11]

For immunohistochemistry analysis, tumors and organs were fixed and embedded in paraffin. The sections of 4 μm thickness were mounted on glass slides. To analyze the proliferative index, tumor sections were incubated for 1 hour at 4°C with the Ki67 antibody (Abcam, Cambridge, UK) with 1:200. Apoptosis was analyzed using the terminal deoxynucleotidyl transferase-mediated dUTP nick-end labeling (TUNEL) assay (TUNEL assay kit-HRP-DAB, Abcam), according to the manufacturer's instructions. Paraffin-embedded tumors were sliced to 4 μm thickness; after deparaffinization and permeabilization, the sections were incubated with proteinase K for 15 minutes at room temperature, then washed three times with PBS. The terminal deoxynucleotidyl transferase (TdT) Labeling Reaction Mix was added and placed in the humidified chamber at room temperature for 1.5 hours to catalyze the addition of biotin-labeled deoxynucleotides to exposed 3'-OH ends of DNA fragments. Streptavidin-horseradish peroxidase conjugate was added to biotin-labeled deoxynucleotides in the samples

for detection. The reaction was stopped, staining was developed by diaminobenzidine (DAB), and counterstaining was performed using a Methyl Green Counterstaining solution. TUNEL staining pictures were acquired with a slide scanner PANNORAMIC SCAN II (3DHISTECH, Budapest, Hungary). For Ki-67 and TUNEL-positive cell quantification, images were randomly selected and zoomed depending on image quality and values that QuPath (version 0.3.2; https://qupath.github.io)[12] automatically proposed. Next, automated image processing and nuclei segmentation passed through supervised machine learning based on 37 parameters assessing the color, texture, and edges; and the creation of binary to eliminate debris pixels and, in the case of non-labeled nuclei, non-tumor fusiform and elongated cells. Tumor tissue areas were calculated after eliminating microscopic field areas corresponding to “no tissue”. Staining of the tumors and organs and quantification were examined by pathologists who were blinded for the treatment conditions.

Intrafemorally injected murine models

NSGA mice (Prkdc, Il2rg Double KO) were purchased from Junga Bio Co., Ltd Company (Seoul, South Korea). All animal studies were performed according to the guidelines provided by the Institute of Animal Care and Use Committee of Seoul National University, Seoul, South Korea. The protocol was approved by the Committee of Ethics of IACUC (SNU-230503-4-1). Mice were injected in their right femur with 2.5 x 10^5 U937Luc or TF-1Luc cells (injection volume = 5 µl), by insertion of a 25G (2x2 gauze) syringe needle through the knee joint of isoflurane gas anesthetized mice on Day 0. [13] On Day 1, we confirmed the engraftment of the AML cells by bioimaging. Subsequently, mice were randomized to the treatment groups (five mice per group) and treated on Day 3 with vehicle (5% DMSO in sterile water), 0.5mg/kg digitoxin, and 0.5mg/kg UNBS1450, according to the scheme of Supplementary Figure S14L, by intraperitoneal injection. Animals were monitored daily and were euthanized upon signs of distress/disease (weight loss >15%, decreased activity, and/or hind limb paralysis). To monitor

leukemia burden, transplanted mice underwent in vivo bioluminescence imaging at various time points, as specified in Supplementary Figure S14, using an IVIS Spectrum (PerkinElmer, Waltham, MA). The bioluminescence imaging (BLI) signal was assessed after intraperitoneal injection of 150 mg/kg D-Luciferin (Perkin Elmer). Mice were anesthetized with vaporized isoflurane and placed in the imaging chamber. After 5min, mice were imaged with an exposure time of 1 min. Bioluminescent image data were recorded on Living Image® software (version 4.8.0, Caliper Life Sciences, Perkin Elmer), with photons detected converted to total flux (photons/sec) and further analyzed using Aura imaging software. Signal intensity was determined by whole-body ROI quantification.

To validate the leukemia burden in the different mouse groups, bone marrow cells from the right femur and tibia were extracted following an established protocol [14]. Residual red blood cells were removed using RBC lysis buffer (BioLegend, Seoul, Korea) before staining with BD Horizon™ Fixable Viability Stain 510 (BD Biosciences, Seoul, Korea). Cells then were washed in 1 x PBS and stained with phycoerythrin (PE)-conjugated anti-human CD45 (hCD45; BD Pharmigen™, San Diego, CA). A BD FACSLyric™ cytometer (BD Biosciences) was used to analyze the hCD45-stained tumor cells in mouse bone marrow cells. Recorded data were further analyzed and quantified using FlowJo software (version 10.8.1, BD Biosciences, La Jolla, CA).

Proteasome activity assay

The proteasomal activity was assessed with the Proteasome-Glo (Chymotrypsin-like/Caspase-like/Trypsin-like) cell-based assays (Promega).

Combinatorial experiments

The AML cell models MOLM-13, OCI-AML3, THP-1, and U937 were cultivated for 6 days in the presence of low concentrations (from 50 to 300nM) of 5-azacytidine (AZA) or 5-aza-2'-deoxycytidine (decitabine; DAC), both purchased from Sigma-Aldrich; then, cells were co-treated with either UNBS1450 (0, 15, 20, 25nM) or digitoxin (0, 25, 50, 100nM) for an additional 18h, before analyzing MMP loss with 100nM MitoTracker® Red (MTR) CMXRos (Molecular Probes, Invitrogen; 30min of incubation at 37°C) using an LSR Fortessa X-20 (Becton Dickinson). The expected responses of the CGs/HMAs combinations were estimated based on the Zero Interaction Potency (ZIP) model using SynergyFinder 3.0 (https://synergyfinder.fimm.fi).[15]

Computational Docking

Initial structures of the Na^+/K^+-ATPase complexed with CGs were obtained from the Protein Data Bank (PDB; PDB ID: 4HYT, 4RES, 4RET)[16,17] and coordinates for UNBS1450 were generated using ChemBioDraw Ultra 14.0 (PerkinElmer, Waltham, MA, USA). After removing ligands in the original data, we performed computational docking using the Autodock Vina program[18] with the protein and the compound as receptor and ligand. Structural superposition of 4HYT, 4RES, and 4RET coordinates was performed using *WinCoot*[19]. Structural representation of docking results was done with PyMOL (The PyMOL Molecular Graphics System, Version 1.8 Schrödinger, LLC., New York, NY, USA).

Bioinformatics analysis

All the raw (.CEL) files were downloaded from the GEO database to process microarray datasets. Each study was normalized separately using the robust multichip average (RMA) implemented in the R package affy.[20] RNAseq datasets were retrieved from different websites; based on the platform, the available format might differ, as listed in Supplementary

Table S17. The count matrix was analyzed with the DEseq2 R package when raw counts were available. For the Beat AML dataset, the $CD34^+$ subgroup includes: the BM CD34-selected sample 17-00053 and the average of the 12 times sequencing of the control BM purified $CD34^+$[21] (extended to 16 times sequencing in the BeatAML2 cohort).[22] CITE-seq was analyzed with the Seurat R package.[23] Briefly, cells were filtered according to the number of detected genes in each cell and mitochondrial reads contamination. The function LogNormalize was used to normalize expression values across cells. A generalized linear model using a Poisson distribution followed by a Tukey post-hoc test was used to assess if gene expression depends on the disease's state.

The connectivity map (CMap) database[24] (https://clue.io/cmap) was interrogated to identify classes of perturbagens giving the most similar transcriptional signature to CGs. Two types of analysis were performed. First, the transcriptional signature of 12 CGs available in the portal collection and included in the class of perturbagens "ATP1A1-targeting ATPase inhibitors" were compared with those of the other classes of perturbagens belonging to the same database. Second, the transcriptional signature generated by UNBS1450 (20nM; 9h of treatment) assessed in a previous study[6] was compared with those of the classes of perturbagens included in Cmap. For this query, we selected the list of genes > 1.5-fold up-regulated or < 0.5-fold downregulated, corresponding to a total of 55 and 24 genes recognized and included in CMap datasets (listed in Supplementary Tables S13-S14). As visualized by the CMap portal after the analysis, the results were captured and shown in Supplementary Fig. S11.

The results published here about pediatric AML patients are, in whole or part, based upon data generated by the Therapeutically Applicable Research to Generate Effective Treatments (https://ocg.cancer.gov/programs/target) initiative, phs000465 (TARGET AML). The data used for this analysis are available at https://portal.gdc.cancer.gov/projects. The

results of the TCGA cohorts are in whole or part based upon data generated by the TCGA Research Network (https://www.cancer.gov/tcga).

Analyses of enriched gene patterns in common with specific tissue/cell-specific signatures (PaGenBase) and Cell Type Signatures (Gene Set Enrichment Analysis, GSEA) and membership enrichment visualization of GO terms indicated were computed with Metascape (https://metascape/org) (21) or gProfiler (https://biit.cs.ut.ee/gprofiler/gost)[25], using as input the differentially up-regulated gene lists in ≥ ATP1A1/BCL2L1 subgroups of TCGA, Beat AML (OHSU), and TARGET-AML cohorts. DGE of TCGA and Beat AML (OHSU) cohorts were retrieved from cBioPortal (https://www.cbioportal.org); the DGE of TARGET-AML was computed using BioConductor R software.

Statistical analysis

Non-parametric ANOVA (Kruskal-Wallis test) was used to assess differences between different FAB groups, while the Mann-Whitney test was used to test pairwise comparison to the overall mean. In the other instances, one or two-way ANOVA followed by appropriate post-hoc tests was applied. Statistics and the number of samples and experiments were detailed in figure legends. P values < 0.05 were considered significant (* < 0.05, ** < 0.01, *** < 0.001, **** < 0.0001). The association between *ATP1A1*/*BCL2L1* expression and clinical features was analyzed using χ^2 and Fisher's exact tests after stratification of AML patients in low and high *ATP1A1*/*BCL2L1* subgroups (respectively, < and ≥ *ATP1A1*/*BCL2L1* expression median).

Supplementary References

1. Mazumder A, Lee JY, Talhi O, Cerella C, Chateauvieux S, Gaigneaux A*, et al.* Hydroxycoumarin OT-55 kills CML cells alone or in synergy with imatinib or Synribo: Involvement of ER stress and DAMP release. *Cancer Lett* 2018 Dec 1; **438:** 197-218.

2. Cerella C, Gaigneaux A, Mazumder A, Lee JY, Saland E, Radogna F*, et al.* Bcl-2 protein family expression pattern determines synergistic pro-apoptotic effects of BH3 mimetics with hemisynthetic cardiac glycoside UNBS1450 in acute myeloid leukemia. *Leukemia* 2017 Mar; **31**(3)**:** 755-759.

3. Radogna F, Cerella C, Gaigneaux A, Christov C, Dicato M, Diederich M. Cell type-dependent ROS and mitophagy response leads to apoptosis or necroptosis in neuroblastoma. *Oncogene* 2016 Jul 21; **35**(29)**:** 3839-3853.

4. Schneider NFZ, Cerella C, Lee JY, Mazumder A, Kim KR, de Carvalho A*, et al.* Cardiac Glycoside Glucoevatromonoside Induces Cancer Type-Specific Cell Death. *Front Pharmacol* 2018; **9:** 70.

5. Chou TC. Drug combination studies and their synergy quantification using the Chou-Talalay method. *Cancer Res* 2010 Jan 15; **70**(2)**:** 440-446.

6. Cerella C, Muller F, Gaigneaux A, Radogna F, Viry E, Chateauvieux S*, et al.* Early downregulation of Mcl-1 regulates apoptosis triggered by cardiac glycoside UNBS1450. *Cell Death Dis* 2015 Jun 11; **6:** e1782.

7. Iamshanova O, Mariot P, Lehen'kyi V, Prevarskaya N. Comparison of fluorescence probes for intracellular sodium imaging in prostate cancer cell lines. *Eur Biophys J* 2016 Oct; **45**(7)**:** 765-777.

8. Nishino M, Dahlberg SE, Cardarella S, Jackman DM, Rabin MS, Hatabu H*, et al.* Tumor volume decrease at 8 weeks is associated with longer survival in EGFR-mutant advanced non-small-cell lung cancer patients treated with EGFR TKI. *J Thorac Oncol* 2013 Aug; **8**(8)**:** 1059-1068.

9. Sutton SK, Carter DR, Kim P, Tan O, Arndt GM, Zhang XD*, et al.* A novel compound which sensitizes BRAF wild-type melanoma cells to vemurafenib in a TRIM16-dependent manner. *Oncotarget* 2016 Aug 9; **7**(32)**:** 52166-52178.

10. Ubezio P. Beyond The T/C Ratio: Old And New Anticancer Activity Scores In Vivo. *Cancer Manag Res* 2019; **11:** 8529-8538.

11. Umbricht CA, Koster U, Bernhardt P, Gracheva N, Johnston K, Schibli R*, et al.* Alpha-PET for Prostate Cancer: Preclinical investigation using (149)Tb-PSMA-617. *Sci Rep* 2019 Nov 28; **9**(1)**:** 17800.

12. Bankhead P, Loughrey MB, Fernandez JA, Dombrowski Y, McArt DG, Dunne PD*, et al.* QuPath: Open source software for digital pathology image analysis. *Sci Rep* 2017 Dec 4; **7**(1)**:** 16878.

13. Cany J, Roeven MWH, Hoogstad-van Evert JS, Hobo W, Maas F, Franco Fernandez R, *et al.* Decitabine enhances targeting of AML cells by CD34(+) progenitor-derived NK cells in NOD/SCID/IL2Rg(null) mice. *Blood* 2018 Jan 11; **131**(2)**:** 202-214.

14. Amend SR, Valkenburg KC, Pienta KJ. Murine Hind Limb Long Bone Dissection and Bone Marrow Isolation. *J Vis Exp* 2016 Apr 14; (110).

15. Ianevski A, Giri AK, Aittokallio T. SynergyFinder 3.0: an interactive analysis and consensus interpretation of multi-drug synergies across multiple samples. *Nucleic Acids Res* 2022 May 17.

16. Laursen M, Yatime L, Nissen P, Fedosova NU. Crystal structure of the high-affinity Na+K+-ATPase-ouabain complex with Mg2+ bound in the cation binding site. *Proc Natl Acad Sci U S A* 2013 Jul 2; **110**(27)**:** 10958-10963.

17. Laursen M, Gregersen JL, Yatime L, Nissen P, Fedosova NU. Structures and characterization of digoxin- and bufalin-bound Na+,K+-ATPase compared with the ouabain-bound complex. *Proc Natl Acad Sci U S A* 2015 Feb 10; **112**(6)**:** 1755-1760.

18. Trott O, Olson AJ. AutoDock Vina: improving the speed and accuracy of docking with a new scoring function, efficient optimization, and multithreading. *J Comput Chem* 2010 Jan 30; **31**(2)**:** 455-461.

19. Emsley P, Lohkamp B, Scott WG, Cowtan K. Features and development of Coot. *Acta Crystallogr D Biol Crystallogr* 2010 Apr; **66**(Pt 4)**:** 486-501.

20. Irizarry RA, Hobbs B, Collin F, Beazer-Barclay YD, Antonellis KJ, Scherf U, Speed TP. Exploration, normalization, and summaries of high density oligonucleotide array probe level data. *Biostatistics* 2003 Apr; **4**(2)**:** 249-264.

21. Tyner JW, Tognon CE, Bottomly D, Wilmot B, Kurtz SE, Savage SL*, et al.* Functional genomic landscape of acute myeloid leukaemia. *Nature* 2018 Oct; **562**(7728)**:** 526-531.

22. Bottomly D, Long N, Schultz AR, Kurtz SE, Tognon CE, Johnson K*, et al.* Integrative analysis of drug response and clinical outcome in acute myeloid leukemia. *Cancer Cell* 2022 Aug 8; **40**(8)**:** 850-864 e859.

23. Butler A, Hoffman P, Smibert P, Papalexi E, Satija R. Integrating single-cell transcriptomic data across different conditions, technologies, and species. *Nat Biotechnol* 2018 Jun; **36**(5)**:** 411-420.

24. Subramanian A, Narayan R, Corsello SM, Peck DD, Natoli TE, Lu X*, et al.* A Next Generation Connectivity Map: L1000 Platform and the First 1,000,000 Profiles. *Cell* 2017 Nov 30; **171**(6)**:** 1437-1452 e1417.

25. Raudvere U, Kolberg L, Kuzmin I, Arak T, Adler P, Peterson H, Vilo J. g:Profiler: a web server for functional enrichment analysis and conversions of gene lists (2019 update). *Nucleic Acids Res* 2019 Jul 2; **47**(W1)**:** W191-W198.

Supplementary Tables legends

Supplementary Table S1. Enrichment of monocytic markers in *ATP1A1/BCL2L1*^{high} AML (≥ median values).

Supplementary Table S2-S4. Enriched GO terms related to the innate immune system in TCGA, Beat AML, and TARGET AML cohorts, respectively.

Supplementary Table S5-S9. Clinical features associated with high *ATP1A1/BCL2L1* ratio (≥ median) in TCGA-AML, Beat AML (OHSU, 2018), Leucegene, Verhaak, and TARGET-AML cohorts.

Supplementary Table S10. List of FAB M4, M5, and M6 AML cell lines selected from the EMBL-EBI ATLAS expression portal.

Supplementary Table S11. List of cell lines included in the CCLE dataset.

Supplementary Table S12. List of cancer types included in the GDC Pan-cancer TCGA (PANCAN) cohort**.**

Supplementary Table S13. List of 55 upregulated genes (> 1.5-fold change) by UNBS1450 in U937 cells.

Supplementary Table S14. List of 24 downregulated genes (< 0.5-fold change) by UNBS1450 in U937 cells.

Supplementary Table S15. The response rates of U937 and Hel mouse xenografts to the CGs UNBS1450 and digitoxin (TGI and REG).

Supplementary Table S16. Relative body weight change in U937 and Hel mouse xenografts treated with the CGs UNBS1450 and digitoxin.

Supplementary Table S17. List of datasets and databases used in this study.

Supplementary Figure Legends

Supplementary Fig. S1. Association between FAB classification and the expression of selected single or combined markers from selected publicly available AML datasets. A. Levels of *ATP1A1*, *BCL2L1*, *ATP1A3*, and *ATP1A3*/*BCL2L1* ratios were analyzed according to FAB subtype classification in the same cohorts of Fig. 1. The analyses were carried out considering the expression levels of the single markers reported (expressed in RPKM for TCGA and Beat AML); log2(FPKM-uq+1) for TARGET AML; log2(FPKM) for Verhaak and Metzeler datasets. Primary *de novo* specimens were selected for the analysis to exclude duplicated values. Kruskal-Wallis test for median comparisons; further comparisons between the median of each subgroup and the overall median (dashed line) were performed applying the Mann-Whitney test (P values: * < 0.05, **< 0.01, ***< 0.001, **** < 0.0001).

Supplementary Fig. S2. Enrichment and cell type signature analyses. A. Summary of enrichment analysis in PaGenBase (pattern of genes in common with specific tissue/cell-specific signature); and **B.** Cell Type Signatures (Gene Set Enrichment Analysis, GSEA) as elaborated by Metascape on the upregulated gene list in high *ATP1A1*/*BCL2L1* ratio (≥ median) of TCGA (N=1802 genes), Beat AML (N=3000 genes), and TARGET-AML (N=2994 genes) cohorts provided as input. **C.** Odds plot of the cell type enrichment in high *ATP1A1*/*BCL2L1* Beat AML and TARGET-AML patients. Red: significantly upregulated genes; blue: significantly downregulated genes; grey: not significant (up or downregulated) genes.

Supplementary Fig. S3. Comparative analysis between healthy mononuclear cells and AML blasts. A. Analysis of *ATP1A1*/*BCL2L1* ratio on BeatAML2 (Vizome) dataset including non-cancerous mononuclear cells. P values: **** < 0.0001, compared with the overall median: Kruskal-Wallis test; further comparisons between the median of each subgroup and the overall

median (dashed line) or specific groups: Mann-Whitney test (P values: ****/#### < 0.0001). **B**. Analysis of *CD14* expression in the same samples of Fig. 1D. Kruskal-Wallis test; Mann-Whitney test for comparisons between the median of each subgroup and the overall median (dashed line) or specific groups (P values: * < 0.05, **** < 0.0001).

Supplementary Fig. S4. Association of *KMT2A*, *CBFB-MYH11*, and *FLT3-TKD* alterations with M4/M5 maturation stage and high *ATP1A1/BCL2L1* expression levels. FAB distribution of (**A**) *CBFB-MYH11* fusion in TCGA LAML and TARGET AML (**C**), *KMT2A* rearrangements (TARGET-AML), and (**E**) *FLT3* mutations (pool of all five AML cohorts). Fisher's exact or Chi-square tests were applied. Levels of ATP1A1/BCL2L1 ratios were analyzed according to the alteration indicated (**B**, **D**, **F**). Kruskal-Wallis test or Mann-Whitney test for comparisons between the median of each subgroup and the overall median (dashed line) or specific groups (P values: * < 0.05, ** < 0.001, *** < 0.001, **** < 0.0001).

Supplementary Fig. S5. Absence of correlation between selected protein expression levels and the cytotoxic response to UNBS1450 *in vitro* and *ex vivo*. A. UNBS1450 AUC values calculated on UNBS1450-treated AML blasts of a cohort of 23 *de novo* AML patients previously investigated.[2] Specimens are in the same order as the previous study. UNBS1450 dose-response curves for each AML patient specimen used for AUC computation. **B.** Quantification of the relative band intensity of the indicated proteins in AML blasts of the first 17 specimens of the original cohort and **C.** correlation analysis between the indicated individual and multifactorial protein expression levels and the UNBS1450 AUC values (N=17; non-parametric one-sided Spearman). Quantification values of MCL1 and BCL2 protein bands on the same AML specimens are from our previous study. [2] **D.** Same analysis as in (**C**). for FAB M1-M2 (top; N=6) vs. FAB M4-M5 (bottom; N=11) AML subtypes. Quantification of the

relative band intensity of the indicated proteins in 14 cell models and correlation analysis between **E**. them or **F**. the ratios between ATP1A1/ATP1A3 and BCL2 or MCL1 proteins and UNBS1450 IC50 mean values (see Fig. 2A-F). Data represent the mean of three independent experiments ± SD. **F.** Differential levels of ATP1A1/BCL2L1 ratio, ATP1A1, and BCL2L1 levels in a list of AML cancer cell lines (see Supplementary Table S10), selected from the EMBL-EBI database, and including FAB subtypes M4, M5, and M6 (Kruskal-Wallis test). Comparisons between the median of each subgroup and the overall median (dashed line; *) or between the medians of specific subgroups ($): Mann-Whitney test (P values: ** < 0.01 with respect to the overall median; $$ < 0.01, M5 vs. M6; ## < 0.01 M4 vs. M5 or M6).

Supplementary Fig. S6. *ATP1A1/BCL2L1* ratio levels across cancer cell types and cell lineages (pan-cancer analysis). Analysis of *ATP1A1* and *BCL2L1* levels in (**A**, **B**) 40 cancer cell lineages and (**C**, **D**) 33 primary cancer types, respectively listed in the CCLE and TCGA pan-cancer datasets (Fig. 2G-H and Supplementary Tables S11-S12). The values for the single gene expression were in log2(RPKM) for CCLE and log2(FPKM)–uq+1) for the TCGA pan-cancer dataset. The bars corresponding to AML are highlighted in red (Kruskal-Wallis test; further comparisons between the median of each subgroup and the overall median (dashed line) with the Mann-Whitney test (P values: * < 0.05, **< 0.01, ***< 0.001, **** < 0.0001).

Supplementary Fig. S7. No impact of CGs on monocytes/lymphocytes from healthy donors. Gating (**A**) and quantification of monocyte (**B**) and lymphocyte (**C**) sub-populations from PBMCs of healthy donors treated with different concentrations of UNBS1450 and digitoxin in FSC-H vs. SSC-H dot plots. Positivity to CD14 was used to confirm monocytes vs. lymphocytes. At least 5 independent experiments; One-way ANOVA, post-hoc: Dunnett). **D**) FSC vs. SSC profiles from selected AML patient blasts representative of different FAB

subtypes, untreated or treated with UNBS1450, compared with the FSC vs. SSC profiles of healthy donors shown in (**A**). The position of the lymphocytic population was ensured upon conventional CD45 vs. SSC analysis and back-gating. The viability of these AML samples was previously assessed by positivity to annexin V/Zombie assay or MitoTracker® Red staining at the FACS upon conventional CD45 vs. SSC gating to exclude non-myeloid components [2].

Supplementary Fig. S8. Consistent protective effect of Na^+ and K^+ modulation on the UNBS1450 cytotoxic effect in myeloid cancer cell lines. A. OCI-AML3 AML cell line was challenged with UNBS1450 after incubation in HBSS-modified media containing 150 or 50mM Na^+ as done for U937 cells (Fig. 3D-F), and mitochondrial membrane potential loss was estimated after MitoTracker® Red staining (30nM UNBS1450, 18h; N=3). B. AML OCI-AML3, THP1, MV4-11, and CML K562 were challenged for 18h with UNBS1450 (30 or 50nM in K562 cells) in a medium supplemented with the indicated concentrations of KCl before proceeding with the mitochondrial membrane potential analysis (N=3). **C**. U937 cells were incubated for one h with the non-specific K^+ channel inhibitor TEA (Sigma-Aldrich) and then challenged with 30nM UNBS1450. After 18h, mitochondrial membrane potential loss (left) and caspase-3 (CASP-3) cleavage (right) were assessed and quantified (N=3). Significant statistical differences are reported between untreated vs. UNBS1450-treated cells (*) and UNBS1450-treated cells in the presence/absence of the modulating condition (# or &). Two-way ANOVA; post-hoc: Sidak or Dunnett; P values: */#/& < 0.05, ##/&& < 0.01, ### < 0.001, ****/####/&&&& < 0.0001).

Supplementary Fig. S9. The analysis of the nuclear morphology confirms the robust sensitization of BCL2L1 overexpressing cells to UNBS1450-induced apoptosis by *BCL2L1* chemical and genetic inhibition. Apoptosis in the same samples of Fig. 4 was assessed in

parallel by quantifying cells with apoptotic nuclear morphology upon Hoechst staining. Representative photos of **A.** Hel, **B.** TF-1, and **C.** CML K562 after single or combined treatment with 100nM ABT-263 and 30nM UNBS1450 (both sub-toxic concentrations). The percentage of the cells with apoptotic fragmented/condensed nuclei is reported on the right.[2, 4, 6, 26] **D.** and **E**. Similarly, the percentage of cells with fragmented/condensed nuclei was monitored and quantified in transfected cells after UNBS1450 treatment (18h, 30nM). All the results correspond to three independent experiments (Two-way ANOVA; post-hoc: Sidak; statistical significance: * < 0.05, * < 0.01, *** < 0.001, **** < 0.0001).

Supplementary Fig. S10. The selective BCL2 inhibitor ABT-199 does not sensitize BCL2L1-overexpressing cells to UNBS1450. Hel, TF-1, and K562 cells were treated with ABT-199 instead of ABT-263 in a single or combinatorial regimen with 50nM UNBS1450 to confirm no modulatory effect of the selective BCL2 inhibition on these cells. Analysis of apoptosis induction in BCL2L1 overexpressing **A.** Hel, **B.** TF-1, and **C.** CML K562 cells by Annexin-V/PI viability assay (BD Pharmingen). N=3; Two-way ANOVA; post-hoc: Sidak.

Supplementary Fig. S11. The similarity between the transcriptional signature of UNBS1450 and protein synthesis inhibitors. A. The list of 12 CGs included in the CMap database[24] gives the most similar gene expression signature to the “ATP1A1-targeting ATPase inhibitor” and “protein synthesis inhibitor” perturbagen classes. **B**. UNBS1450 shares the most similar transcription signature to the same perturbagen classes. For this query, we selected the list of genes > 1.5-fold upregulated or < 0.5-fold downregulated upon 9h of treatment with 20nM UNBS1450, corresponding to a total of 55 and 24 genes recognized and included in CMap datasets (see Supplementary Tables S13-S14, and our previous study[6]). Top-list categories with the highest similarity score are ordered in descending order in both panels.

Supplementary Fig. S12. Protein synthesis inhibition is a hallmark of CGs. A. MCL1, MYC, CCND1, and CASP-3 protein band quantification at progressive treatment times of U937 cells with UNBS1450 (see Fig. 5A). **B**. The efficiency of protein synthesis was further analyzed in AML THP-1 and Hel cells, the latter more resistant to UNBS1450. In parallel, the modulation of MCL1 was assessed (bottom panels). The effect of UNBS1450 was compared with digitoxin (8h of treatment) and two protein synthesis inhibitors, the prototypical cycloheximide (CHX; 10µM, 4h) and the clinically approved homoharringtonine (HHT; 4h). C. Same analysis on PHA/IL-stimulated proliferating PBMCs. **D**. No modulation of mTOR mediators in U937 cells (25nM UNBS1450, 8h treatment); the mTOR inhibitor PP242 (10µM, 8h; Sigma-Aldrich) was used as control (N=3) **E**. Absence of proteasome modulation in the same conditions of Fig. S12D (N=3). **F**. MCL1/BCL2 correlation analysis with UNBS1450 AUC values (top) and FAB stratification of MCL1/BCL2, BCL2, MCL1, and BCL2L1. (N=17; non-parametric one-sided Spearman). **G**. Correlation studies between MCL1/BCL2L1 and MCL1/BCL2 and UNBS1450 AUC values on restricted FAB M1-M2 (N=6) vs. FAB M4-M5 (N=11) AML blasts. **H**. MCL1/BCL2L1 and MCL1/BCL2 correlation studies in established cell lines (N=14). MCL1 and BCL2 values were previously analyzed and retrieved for correlation studies.[2]

Supplementary Fig. S13. Myelomonocytic and monocytic AML express higher *MCL1/BCL2L1* ratio levels. FAB distribution of **A.** *MCL1/BCL2L1* ratio and **B.** *MCL1* expression levels in the same cohorts of Fig.1. Kruskal-Wallis test for median comparison; Mann-Whitney test for further comparisons between the median of each subgroup and the overall median (dashed line; P values: * < 0.05, **< 0.01, ***< 0.001, **** < 0.0001). Primary *de novo* specimens in all datasets were only selected. **C.** Differential *MCL1/BCL2L1* ratio and

MCL1 levels in the AML cancer cell lines listed in the EMBL-EBI database, including FAB subtypes M4, M5, and M6 (see Supplementary Table S10; Kruskal-Wallis test). Comparisons between the median of each subgroup and the overall median (dashed line; *) or between the medians of specific subgroups ($) were determined by the Mann-Whitney test (P values: ** < 0.01 (with respect to the overall median); $$ < 0.01 (M5 vs. M6); ## < 0.01 M4 vs. M5 or M6). **D**. 11 pairs of diagnostic vs. relapse AML specimens after conventional chemotherapy from Hackl et al., GSE66525[27]; **E**. phenotypically primitive vs. monocytic *de novo* AML at the diagnosis vs. the relapsed clones present in AML patient 12, retrieved from Pei et al.[28] (CITE-seq, GSE143363). Mann-Whitney assay (P values: * < 0.05, **< 0.01, ***< 0.001, **** < 0.0001).

Supplementary Fig. S14. Impact of the CG UNBS1450 and digitoxin on human M5 vs. M6 AML cell line-derived xenografts. A. Treatment plan for mice xenografts for experiments I. and II (Fig. 6A-B and 6C-D). Days of treatment/tumor and organ collection are indicated in: blue for U937, green for Hel, and black when the days were common to both xenografts. **B.** Confirmatory analysis of expression levels of the major anti-apoptotic BCL2 proteins in Hel and U937 cells before mice injections. **C.** The comparable mean tumor volume reached in the vehicle group of both xenografts in experiments I and II defined the end of the study for each xenograft model. **D.** Images of each treatment group (1.0mg/Kg UNBS1450 or digitoxin; vehicle) of the sacrificed BALB/c mice xenografted with M5 U937 (top) and M6 Hel (bottom) from the experiment I. The percentage of tumor volume (mm^3) changes with the time in each treatment group in M5 U937 (top) and M6 Hel (bottom). Relative tumor volume change induced by digitoxin or UNBS1450 for the vehicle-treated group in U937 vs. Hel xenografts in **E.** experiment I, **F.** experiment II. **G.** Summary of the response type in U937 and Hel xenografts at the endpoints (TGI: tumor growth inhibition; REG: regression); see Suppl. Table

XIV for all measurement times. When the tumor volume increased in treated samples with respect to the treatment start (ΔT=T-T0 ≥ 0), the impact of CGs on tumor growth was compared to those of the vehicle group and estimated as the percentage of TGI; when the tumor volume decreased in treated samples with respect to the treatment start (ΔT=T-T0 < 0), tumor regression was estimated as specified in methods.[10] **H-I.** Ki-67 and TUNEL staining of U937 and Hel tumor tissues with quantification (right panels). **J-K.** Body weight at any time-point of experiments I and II. All data correspond to the mean ± SEM. Dashed line: relative tumor volume change at T0. TGI: tumor growth inhibition; REG: tumor regression. **L.** Treatment plan for U937-Luc and TF-1 mice xenografts shown in Figure 6K-O. **M.** Kinetic analysis of the bioluminescence and **N.** body weight estimated at the indicated days. Statistical analysis: **E-F-J-K-M-N**: Two-Way ANOVA; post-hoc: Tukey or Sidak (trend); and Dunnett (comparison at the endpoint between groups). **H-I**: One-way ANOVA; Kruskal-Wallis, post-hoc: Dunn's (Ki-67 and Tunel staining). P values: */# < 0.05, **/##< 0.01, ***/< 0.001, ****/[$$$$] < 0.0001.

Supplementary Fig. S15. Potential combinatorial regimens including CGs. A. Heatmap of the cell drug-cell type correlation, ordered by and limited to correlations significant in monocyte-like cells, extracted from the BeatAML2 cohort[21] (http://vizome.org/aml2/). **B.** Correlation analysis between VEN resistance (AUC values) and *ATP1A1*/*BCL2L1* expression from the same cohort (N=389; non-parametric one-sided Spearman). **C**. Heatmap of the synergism score between UNBS1450 or digitoxin and the HMAs 5'-azacytidine (AZA) or decitabine (DAC) calculated with SynergyFinder3.0 (ZIP model).[15] **D.** Correlation analysis between AZA resistance (AUC values) and *ATP1A1*/*BCL2L1* expression from BeatAML2 cohort (N=387; non-parametric one-sided Spearman).

Supplementary Fig. S16. UNBS1450-treated BCL2L1 overexpressing cells undergo specific alterations of the cell cycle and cell morphology. A. Flow cytometric profile and **B.** analysis of the cell cycle in M5 U937 at progressive treatment times with UNBS1450. **C**. Flow cytometric profile of BCL2L1 overexpressing M6 Hel cells treated/untreated with UNBS1450 (30nM; 24h). **D**. In situ analysis of the nuclear/cellular morphology of the same samples **E.** Cell cycle analysis after 24h and 48h treatment of Hel with 0, 30, or 50mM UNBS1450 (N=3). The same analysis was extended to the other BCL2L1 overexpressing M6 TF-1 (**F.**, **G.**, **H.**) and CML K562 (I., **J.**, **K.**) cells (N=3). Statistical significance of the changes occurring in each cell cycle phase was estimated (Two-way ANOVA; post-hoc: Dunnett; P values: *< 0.05, ** < 0.01, ***< 0.001, ****< 0.0001).

Supplementary Fig. S17. Correlation between RNA and protein expression data for the markers of interest. A. Computed Pearson and Spearman correlation values for the indicated genes as available from Nusinow et al.[29] Correlation between mRNA expression level (log2(TPM+1); 22Q1 Public) and relative protein expression (proteomics) values computed by and retrieved from the Depmap portal (https://depmap.org/portal/) for **B.** ATP1A1 and **C.** BCL2L1, based on the list of blood cell lines available in the database and indicated in **D**.
